# Circular RNA CircFOXO3 Functions as a Competitive Endogenous RNA for Acid-Sensing Ion Channel Subunit 1 Mediating Oxeiptosis in Nucleus Pulposus

**DOI:** 10.3390/biomedicines12030678

**Published:** 2024-03-18

**Authors:** Xi Chen, Ying Song, Guanghui Chen, Baoliang Zhang, Yang Bai, Chuiguo Sun, Dongwei Fan, Zhongqiang Chen

**Affiliations:** 1 Department of Orthopaedics, Peking University Third Hospital, Beijing 100191, China; chenxi29311@163.com (X.C.); chenguanghui@bjmu.edu.cn (G.C.); sunchuiguo@163.com (C.S.); fdw@bjmu.edu.cn (D.F.); 2 Beijing Key Laboratory of Spinal Disease Research, Beijing 100191, China; 3 Engineering Research Center of Bone and Joint Precision Medicine, Ministry of Education, Beijing 100191, China; 4 Center of Basic Medical Research, Institute of Medical Innovation and Research, Peking University Third Hospital, Beijing 100191, China; yingsong@hsc.pku.edu.cn (Y.S.); yangbxx2019@163.com (Y.B.)

**Keywords:** intervertebral disc degeneration, oxeiptosis, circular RNA, microRNA, ASIC1, in vitro, in vivo

## Abstract

Oxeiptosis is a reactive oxygen species (ROS)-induced pathway of cell death. The involvement of circular RNAs (circRNAs) has been confirmed in the incidence and progression of intervertebral disc degeneration (IVDD). However, whether oxeiptosis occurs in IVDD and how circRNAs regulate oxeiptosis is still unclear. In this study, we discovered that oxeiptosis could be induced in nucleus pulposus cells (NPCs), and circFOXO3 was significantly upregulated after oxeiptosis induction. Transfection using circFOXO3 small interfering RNA (siRNA) significantly inhibited oxeiptosis in NPCs. Mechanistically, circFOXO3 upregulated acid-sensing ion channel subunit 1 (ASIC1) expression by functioning as a molecular sponge for miR-185-3p and miR-939-5p. Subsequent rescue experiments validated that circFOXO3 could regulate oxeiptosis in NPCs via the miR-185-3p/miR-939-5p-ASIC1 axis. Further research on ASIC1 functions indicated that this regulation was achieved by affecting the Calcium ion (Ca^2+^) influx mediated by ASIC1. A mouse IVDD model was established, and silencing circFOXO3 in vivo was found to inhibit IVDD development and the activation of the oxeiptosis-related pathway. Overall, circFOXO3 is one of the factors contributing to the progression of IVDD by mediating oxeiptosis.

## 1. Introduction

Low back pain stands as a primary reason for lower limb movement disorders or disability globally, with lumbar intervertebral disc degeneration (IVDD) being identified as a significant underlying reason, mainly caused by obesity, wrong nutrition profile, lack of fitness activities, and trauma (incidental single-event injury or prolonged wear and tear) [1,2,3,4]. Due to the paucity of vascular distribution in an intervertebral disc (IVD), its metabolic activity relies on the free diffusion of the cartilage endplate. As mechanical and chemical damage accumulates and IVDD develops, the efficiency of this free exchange diminishes, leading to respiratory dysfunction and mitochondrial damage of nucleus pulposus cells (NPCs) and causing a rise in the intracellular reactive oxygen species (ROS) level. This, in turn, further hampers the efficiency of free exchange and worsens IVDD [5,6]. However, the mechanism by which toxic levels of ROS lead to damage and death of NPCs in IVDs is currently unclear.

Oxeiptosis, a caspase-independent, ROS-sensitive, and non-inflammatory cell death pathway, is triggered and mediated by the stimulation of the Kelch-like ECH-associated protein 1 (KEAP1)–phosphoglycerate mutase family member 5 (PGAM5)–apoptosis-inducing factor mitochondrion-associated 1 (AIFM1) pathway [7]. KEAP1 is an important oxidative stress sensor that can specifically respond to intracellular levels of ROS [8]. At moderate levels of ROS, KEAP1 releases and reduces the ubiquitination-mediated degradation of nuclear factor erythroid 2-related factor 2 (Nrf2), exerting a protective effect against oxidative stress [8,9]. However, under toxic levels of ROS, KEAP1 dissociates from PGAM5, and PGAM5 is internalized into mitochondria, resulting in the dephosphorylation of a serine residue at 116 of AIFM1, thereby initiating oxeiptosis [7,9,10]. Inhibition of oxeiptosis has been shown to attenuate the reduced viability caused by ROS treatment [11,12,13,14,15]. Based on these findings, we wondered if oxeiptosis is induced in NPCs and whether inhibiting oxeiptosis in NPCs could delay the progression of IVDD.

Circular RNA (circRNA) is a distinct type of RNA that forms a circular construction by linking the 5′ and 3′ ends in a head-to-tail manner. Recent research has highlighted circRNAs role as microRNA (miRNA) sponges (a kind of competitive endogenous RNA mechanism) [16,17,18,19,20]. One particular circRNA of interest is circFOXO3, which originates from the back-splicing of the second exon of the *forkhead box O3* (*FOXO3*) gene. CircFOXO3 has been found to regulate ROS-related cellular activities in various cell types, tissues, and cancer. In these reports, the silencing of circFOXO3 attenuated the impact of ROS in vitro and in vivo [21,22,23,24,25,26,27]. This experimental evidence indicated that inhibiting circFOXO3 holds the potential for suppressing ROS-induced oxeiptosis in NPCs.

In the present study, we observed ROS-induced oxeiptosis in NPCs both in vitro and in vivo. Additionally, we elucidated the circFOXO3-miR-185-3p/miR-939-5p-acid-sensing ion channel subunit 1 (ASIC1) axis presence, which has a vital function in modulating the susceptibility of NPCs to oxeiptosis. Notably, the effective silencing of circFOXO3 leads to the inhibition of oxeiptosis and contributes to the delay in IVDD progression.

## 2. Materials and Methods

### 2.1. Ethics Statement

This study was permitted and directed by the Ethics Committee of Peking University Third Hospital (No. M2021317 for humans; No. A2022127 for animals). Human nucleus pulposus (NP) tissues were acquired from patients undergoing an operation at the Department of Orthopaedics of Peking University Third Hospital. Before tissue collection, all patients were informed about the nature of the operations and the associated risks, and they granted written informed consent for the utilization of their tissue samples for scientific objectives.

### 2.2. CircRNA Microarray and Bioinformatics Analysis

The Gene Expression Omnibus (GEO) database was employed to acquire circRNA expression data (accession number GSE67566). The “normalizeBetweenArrays” function from the LIMMA package was employed to normalize raw data. Differential expression analysis between the IVDD and control samples was performed using the linear model of the LIMMA package. The obtained *p*-values were then adjusted utilizing the False Discovery Rate correction, and the thresholds for identifying differentially expressed circRNAs were established at a fold change (FC) of log_2_FC ≥ 0.58 or ≤−0.58, and a *p*-value < 0.05.

The complete sequences of miRNAs and circFOXO3 (hsa_circ_0006404/mmu_circ_0002207) were acquired from miRBase (https://www.mirbase.org/index.shtml, accessed on 20 August 2022) and circBase (http://www.circbase.org/, accessed on 20 August 2022), respectively. We utilized online databases, namely StarBase (https://starbase.sysu.edu.cn/, accessed on 20 August 2022), CircInteractome (https://circinteractome.nia.nih.gov/index.html, accessed on 20 August 2022), and miRanda software (version 3.3a, http://www.microrna.org/, accessed on 20 August 2022), to identify the target miRNAs of circFOXO3 and their corresponding binding sites. Furthermore, TargetScan (http://www.targetscan.org/, accessed on 11 January 2023) online databases were utilized to determine the miR-185-3p and miR-939-5p target genes.

### 2.3. Clinical Samples

We collected a total of 20 samples. Human degenerated NP samples were obtained from 10 patients diagnosed with lumbar IVDD who received discectomy surgery. The normal human NP tissues were acquired from 10 patients with lumbar fractures, matched for age and sex, had no history of IVDD, and underwent decompression and fusion surgery due to neurological deficits associated with the fractures.

### 2.4. Isolation and Culture of Human NPCs

NPCs were extracted from NP tissues by a process of 4 h incubation at 37 °C with a 0.2% solution of type II collagenase in Dulbecco’s modified Eagle medium nutrient mixture F-12 (DMEM/F-12). After isolation, DMEM/F-12, which contained 15% fetal bovine serum, 100 mg/mL streptomycin, and 100 U/mL penicillin, was employed to resuspend the NPCs. They were then incubated in a humidified atmosphere at 37 °C with 5% CO_2_. The cells within the fifth passage were used for subsequent experiments. The transfection of oligo RNAs was carried out using Lipofectamine^™^ 3000 reagents (#L3000008, ThermoFisher Scientific, Invitrogen, Carlsbad, CA, USA) according to the recommended conditions in the user manual.

### 2.5. Adenovirus and Adeno-Associated Virus (AAV) Construction

Adenoviral and AAV vectors were formed and manufactured by Hanbio Co., Ltd. (Shanghai, China). For the adenoviral vectors, overexpression plasmids expressing hsa_circFOXO3 (pAdEasy-EF1-circFOXO3-CMV-EGFP) (Figure S3D) and negative control plasmids were constructed by recombination with an auxiliary packaging plasmid and were then packaged in HEK-293A cells following the manufacturer’s protocol.

As for the AAV vectors, the mouse gene knockdown plasmid (pAAV6-U6-sh_circFOXO3) and the control vector were constructed. Recombinant AAVs were produced through a triple-plasmid transfection system in HEK-293 cells. The specific sh_circFOXO3 sequence used is listed in Table S4. The mouse sh_circFOXO3 sequence was obtained from the work of Yang et al. [25], and the design of the vector and AAV serotype was obtained from the work of Kim et al. [28].

### 2.6. Surgical Procedures and In Vivo Transduction

All mice utilized in this study were acquired from HFK Bioscience Co., Ltd. (Beijing, China). Thirty-six male C57Bl/6 mice, 8 weeks old, were randomly divided into three groups: the sham, IVDD + AAV-sh_NC, and IVDD + AAV-sh_circFOXO3 groups, with 12 mice per group.

Briefly, after the tail was cleaned with alcohol and the corresponding surgical area was exposed, a 30 G syringe needle linked to a microliter syringe was used to puncture the annulus fibrosus (AF) of the Co6/7 (coccygeal 6/7) disc unilaterally about 1.5 mm. The needle then underwent a 180° axial rotation and was left in place for 10 s before slowly injecting 3.0 μL of AAV-6 with a concentration of 10^12^ GC/mL into the disc. The optimal injection volume and dose of AAV were based on previous findings reported by Kim et al. [28]. Upon reaching the end of the fourth week after surgery, the coccygeal vertebrae were isolated from the sacrificed mice for additional analysis.

### 2.7. RNA Extraction and Quantitative PCR (qPCR)

Total cellular RNA was extracted from cultivated NPCs or NP tissues using TRIzol^™^ reagent (#15596, ThermoFisher Scientific, Invitrogen, Carlsbad, CA, USA), following the directions of the manufacturer. After RNA extraction, reverse transcription was conducted, employing 500 ng of total RNA. The Evo M-MLV RT Premix (#AG11706, Accurate Biology, Changsha, China) was employed for complementary DNA (cDNA) synthesis. The miRNA first strand cDNA synthesis kit (#AG11717, Accurate Biology, Changsha, China) was utilized for reverse transcription to study miRNA expression. SYBR^®^ Green Premix Pro Taq HS qPCR Kit (#AG11706, Accurate Biology, Changsha, China) was employed to assess RNA expression levels.

Quantitative PCR was performed in a 20 μL reaction volume. The following were the conditions of the PCR reaction: pre-denaturation for 30 s at 95 °C, followed by denaturation for 5 s at 95 °C and annealing/extension for 30 s at 60 °C, repeated for 40 cycles on a QuantStudio5 system (ThermoFisher Scientific, Applied Biosystems, Carlsbad, CA, USA). To normalize the circFOXO3 and mRNA expression levels, GAPDH was used as a reference, while for miRNAs, U6 served as the reference. The 2^−ΔΔCt^ algorithm was used to calculate the relative expression of each RNA. All primers used in this study are illustrated in Table S1.

### 2.8. Western Blotting

Radioimmunoprecipitation assay lysis buffer (#P0013B, Beyotime, Shanghai, China), which comprises cocktails of protease and phosphatase suppressors (#P1045, Beyotime, Shanghai, China), was used for lysis of the cells/tissues. The Bicinchoninic Acid Protein Assay kit (#C503021, Sangon Biotech, Shanghai, China) was utilized to quantify every protein sample. Equal quantities of protein samples were then separated by 10% sodium dodecyl sulfate–polyacrylamide gel electrophoresis and transferred to Polyvinylidene difluoride (PVDF) membranes. The PVDF membranes were subjected to overnight incubation with primary antibodies against GAPDH (1:1000; #5174T, Cell Signaling Technology, Danvers, MA, USA), AIFM1 (1:1000; #5318, Cell Signaling Technology, Danvers, MA, USA), AIFM1 (Ser-116) phospho-specific (1:1000; #AP5501, ECM Biosciences, Versailles, KY, USA), KEAP1 (1:1000 for human; 1:500 for mouse; #8047, Cell Signaling Technology, Danvers, MA, USA), PGAM5 (1:1000, #ab126534, Abcam, Cambridge, UK, for human; 1:500, #A16022, ABclonal, Wuhan, China, for mouse), ASIC1 (1:1000 for human; 1:500 for mouse; #ab205387, Abcam, Cambridge, UK), matrix metalloproteinase 13 (MMP13) (1:500; #18165-1-AP, Proteintech Group, Wuhan, China), Collagen II (1:500; #28459-1-AP, Proteintech Group, Wuhan, China), Aggrecan (1:500; #13880-1-AP, Proteintech Group, Wuhan, China), and a disintegrin and metalloproteinase with thrombospondin motifs 5 (ADAMTS5) (1:500; #DF13268, AffinitY Biosciences, Liyang, China) at 4 °C and further incubated for 2 h with secondary antibodies at room temperature. Blots were developed using a highly sensitive Plus ECL luminescence reagent (#C510045, Sangon Biotech, Shanghai, China). Detection was performed using the iBrightCL1000 Imaging System (ThermoFisher Scientific, Carlsbad, CA, USA) and quantified using ImageJ software (version 1.53q, National Institutes of Health, USA).

### 2.9. Cell Viability Assay

The cell counting kit-8 (CCK-8) assay was utilized to evaluate the cell viability. Concisely, 96-well plates were utilized for seeding of NPCs, and after the respective treatments, cells were exposed to 1 h incubation with 100 μL of fresh medium containing 10 μL of CCK-8 solution (#C0037, Beyotime, Shanghai, China) at 37 °C. The optical density was detected at 450 nm using the SpectraMax iD3 (Molecular Devices, Sunnyvale, CA, USA).

### 2.10. Annexin V/Propidium Iodide (PI) Staining Assay

The Annexin V-FITC/PI Kit (#KGA108, KeyGEN BioTECH, Nanjing, China) was used to determine the rate of cell death. In summary, 2 × 10^5^ NPCs were collected and then resuspended in a binding buffer with a volume of 200 μL. This was followed by the addition of 2 μL of Annexin V-FITC and 2 μL of PI. The mixture was subjected to 10 min of incubation at room temperature under conditions of darkness. The measurement of cell death rates was conducted using a Cytoflex flow cytometer (Beckman Coulter, Brea, CA, USA). Subsequently, the obtained data were analyzed using the CytExport software (version 2.5.0.77, Beckman Coulter, USA).

### 2.11. ROS Detection Assay

Following the manufacturer’s directions, an ROS Assay Kit (#O040, LABLEAD, Beijing, China) was used to measure cellular ROS levels. In this assay, 5 × 10^5^ NPCs were collected and subjected to incubation with 2′,7′-Dichlorodihydrofluorescein diacetate (DCFH-DA) and serum-free medium solution (1:5000) at 37 °C for 30 min. After three rinses with phosphate buffer saline (PBS), flow cytometry was conducted to analyze ROS levels.

### 2.12. Quantification of Mitochondrial Membrane Potential (MMP; ΔΨm)

A JC-1 Mitochondrial Membrane Potential Assay Kit (#J2220, LABLEAD, Beijing, China) was used to assess MMP. After the treatment of NPCs under the specified conditions, 5 × 10^5^ NPCs were collected and incubated with a diluted JC-1 probe and complete medium at 37 °C for 20 min. Following three rinses and resuspension in the staining buffer, flow cytometry was utilized to analyze cells, and changes in MMP were calculated as the ratio of red (aggregates) to green (monomers) fluorescence intensity.

Mitotracker Red staining was conducted to identify alterations in MMP. After three washes with PBS, a Mitotracker^™^ Red CMXRos (#M7512, ThermoFisher Scientific, Carlsbad, CA, USA) and serum-free medium solution (1:4000) was added, and the cells were subjected to incubation in the dark at 37 °C for 30 min, while nuclei were stained with Hoechst 33342 (#C0030, Solarbio, Beijing, China). A laser confocal microscope (Carl Zeiss, #LSM900, Oberkochen, Germany) was utilized to capture cell images, and the intensity of the mean fluorescence of Mitotracker Red was quantified using ImageJ software (version 1.53q, National Institutes of Health, USA).

### 2.13. Immunofluorescence Assay

The NPCs were fixed and then blocked for 1 h with 1 × PBS/5% normal goat serum/0.3% Triton X-100 buffer. Next, at 4 °C, the cells were incubated overnight with primary antibodies (KEAP1, Rabbit mAb, 1:200; PGAM5, Rabbit mAb, 1:200; COX IV, Mouse mAb, 1:200, #11967, Cell Signaling Technology, Danvers, MA, USA) and incubated with the corresponding secondary antibodies (1:500, #ZF-0511, ZSGB-BIO, Beijing, China; 1:500, #ZF-0513, ZSGB-BIO, Beijing, China). The 4′,6-diamidino-2-phenylindole (DAPI) (#B1061, Applygen Technologies, Beijing, China) was utilized to stain the nuclei. The fluorescence signals were detected using a laser confocal microscope.

### 2.14. Transmission Electron Microscopy (TEM)

NPCs were sequentially fixed by 2.5% glutaraldehyde and then 1% osmic acid in 0.1 M PBS (pH 7.4), and then underwent dehydration using a series of concentrations from 50% to 95% to 100% ethanol. After infiltration, embedding, and polymerizing, ultrathin sections (70 nm) were prepared using a microtome (Leica, #EM UC7, Wetzlar, Germany) with a diamond knife (Diatome, #DU4530, Nidau, Switzerland). The slices underwent a double-staining process employing uranyl acetate and lead citrate for 15 min each, followed by overnight drying. Lastly, a transmission electron microscope (Hitachi, #HT7800, Tokyo, Japan) was used to observe and photograph the slices.

### 2.15. RNA Fluorescence In Situ Hybridization (RNA FISH)

The Cy3-labeled circFOXO3 FISH probe, manufactured by Sangon Biotech Co., Ltd. (Shanghai, China), was designed to specifically detect the “head-to-tail” splicing junction of circFOXO3. The probe sequence is listed in Table S4. Nuclei were counterstained with DAPI. Images were acquired using a laser confocal microscope.

### 2.16. RNA Immunoprecipitation (RIP) Assay

RIP was performed using the Imprint^®^ RNA Immunoprecipitation Kit (#RIP-12RXN, Sigma-Aldrich, St Louis, MO, USA) based on the direction of the manufacturer. Briefly, NPCs were gathered and subjected to lysing. The supernatant of the lysates was then incubated with immunoprecipitation buffer containing antibodies of Argonaute-2 (anti-AGO2; #SAB4200085, Sigma-Aldrich, St Louis, MO, USA) or Immunoglobulin G (anti-IgG; #I4131, Sigma-Aldrich, St Louis, MO, USA) as a negative control. The antibodies were pre-bound on Protein A magnetic beads using a bridging antibody (#R9255, Sigma-Aldrich, St Louis, MO, USA). Following the overnight incubation at 4 °C, TRIzol^™^ reagent was utilized to extract the immunoprecipitated RNA, and the levels of circFOXO3, miR-185-3p, miR-939-5p, GAPDH, and U6 were detected by qPCR.

### 2.17. RNA Pull-Down Assay

Transfection of biotin-labeled hsa-miR-185-3p/hsa-miR-939-5p mimic or mutant (Figure S5B) was performed when NPCs reached more than 70% confluence. The cells were harvested and subjected to lysing after 24 h. Biotin-blocked Streptavidin Magnetic Beads (#P2151; Beyotime, Shanghai, China) were introduced to the lysates and incubated at 4 °C for 6 h. After being rinsed and resuspended, RNA was extracted using TRIzol^™^ reagent. The qPCR was conducted to detect the circFOXO3 and GAPDH levels.

### 2.18. Dual-Luciferase Reporter Gene Assay

Wild-type/mutant (WT/Mut) luciferase reporter plasmids were constructed by Sangon Biotech Co., Ltd. PCR was utilized to amplify a fragment containing the predicted matching sequences of circFOXO3 (Figure S5C) or the 3′UTR of ASIC1 (Figure S5D), and then the amplified fragment was cloned into the psi-CHECK-2 luciferase reporter vector. For the luciferase assay, Lipofectamine^™^ 3000 reagent was employed to co-transfect the HEK-293T cells with the corresponding luciferase reporter plasmid and miRNA mimic or negative control. The cells were lysed and harvested 48 h following the transfection, and luciferase activity was assayed using the Dual-Luciferase Reporter Gene Assay Kit (#EE608001, Sangon Biotech, Shanghai, China) depending on the manufacturer’s instructions.

### 2.19. Intracellular Calcium Ion (Ca^2+^) Measurement

The intracellular Ca^2+^ levels of NPCs were determined using the Fluo-4 Calcium Assay Kit (#S1061, Beyotime, Shanghai, China). After the cells were washed with PBS following the corresponding treatment, the diluted Fluo-4-AM indicator was added, and the cells were incubated at 37 °C in the dark for 30 min. Subsequently, a confocal microscope was utilized to observe the fluorescence and capture images. The mean fluorescence intensity of Fluo-4 was measured using ImageJ software (version 1.53q, National Institutes of Health, New York, NY, USA).

### 2.20. Magnetic Resonance Imaging (MRI) Analyses

Mice from each group underwent an MRI scan using a Discovery 750 3.0 T magnetic resonance scanner (General Electric Medical System, Milwaukee, WI, USA) equipped with a dedicated coil at the end of the fourth week following the surgery. The T2-weighted imaging sequence of the sagittal position of the IVDs was obtained, and the degenerative IVDs were graded by the Pfirrmann grading standard.

### 2.21. In Vivo Bioluminescence Imaging

Mice received AAV–firefly luciferase (fLuc) injections and were subjected to real-time in vivo imaging of bioluminescence. After the injection of 50 μL fluorescein, the bioluminescence was imaged using an in vivo imaging system (VISQUE, Invivo Smart-LF, Anyang, Korea). The imaging was performed at the end of the first and fourth week after the operation.

### 2.22. Histological Evaluations and Terminal Deoxynucleotidyl Transferase dUTP Nick End Labeling (TUNEL) Staining

The specimens of coccygeal vertebrae were collected, fixed with a 4% paraformaldehyde solution for 72 h, and then subjected to decalcification using a 10% ethylenediaminetetraacetic acid solution for 3 weeks. After being embedded in paraffin, the specimens were mid-sagittally sectioned to a 4 µm thickness and subjected to hematoxylin and eosin (H&E) (#G1120, Solarbio, Beijing, China) or Safranin O-Fast Green (#G1371, Solarbio, Beijing, China) staining, and TUNEL staining was conducted utilizing a TUNEL assay kit (#C1086, Beyotime, Shanghai, China) to detect NPC death rate of coccygeal disc specimens based on the manufacturer’s directions. An ECLIPSE CI microscope (Nikon, Tokyo, Japan) was utilized to analyze the histological images. The histological and reference system used is comparable to that reported by Tam et al. [29].

### 2.23. Immunohistochemistry (IHC)

After rehydration, a 3% hydrogen peroxide solution was used to quench endogenous peroxidases. Then, citrate buffer was used for antigen retrieval of the tissue sections, and 5% bovine albumin was used to block the nonspecific protein binding sites. Staining was performed using a primary antibody against ASIC1 (1:200) at 4 °C overnight. On the following day, at 37 °C, the sections were subjected to horseradish peroxidase-conjugated secondary antibody (#PV-9003, ZSGB-BIO, Beijing, China) incubation for 30 min. Then, a diaminobenzidine chromogenic kit (#ZLI-9019, ZSGB-BIO, Beijing, China) was used to visualize the immunoreactivity and hematoxylin for nuclei staining. The slides were visualized under a microscope.

### 2.24. Statistical Analysis

Statistical analysis was performed using the Prism 9 software (Version 9.5.1, GraphPad Software, USA). A two-tailed unpaired Student’s *t*-test was utilized to compare the two groups, and for multiple groups, a one-way analysis of variance was employed. Bonferroni correction was conducted to establish multiple comparisons. A *p*-value less than 0.05 was statistically significant. Every assay was performed in three or more replicates.

## 3. Results

### 3.1. Degenerative NP Tissues Express a Higher Level of circFOXO3

**Figure 1:**
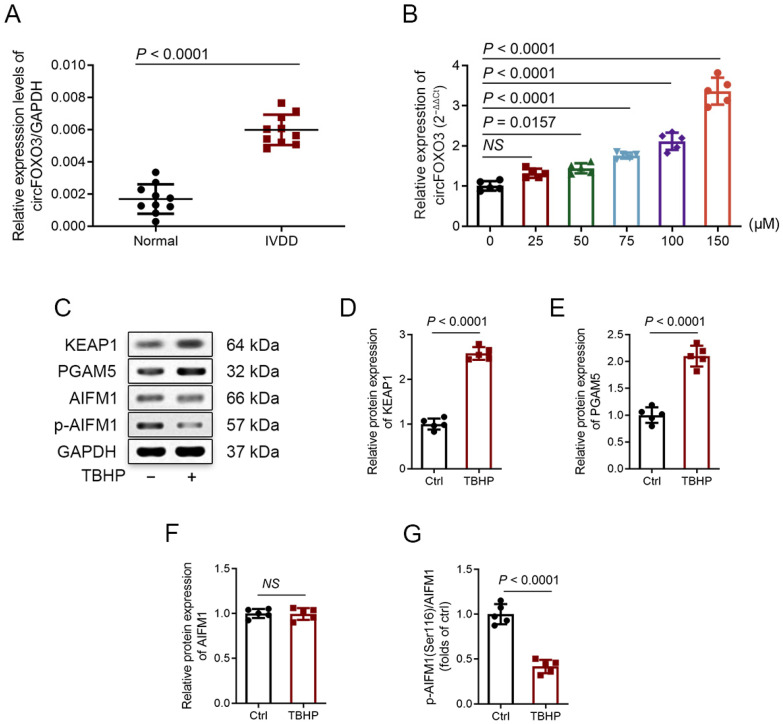
CircFOXO3 detection and expression in nucleus pulposus (NP) tissues and cells; oxeiptosis is induced in *tert*-butyl hydroperoxide (TBHP)-treated nucleus pulposus cells (NPCs). (**A**) CircFOXO3 transcript levels were significantly higher in intervertebral disc degeneration (IVDD) NP tissues than in normal NP tissues (IVDD vs. normal controls: *n* = 10, respectively). (**B**) Time-course analysis of circFOXO3 relative expression in NPCs treated with TBHP (#458139, Sigma-Aldrich, St Louis, MO, USA). (**C**–**G**) Western blot assay of protein expression of KEAP1, PGAM5, and AIFM1 phosphorylated at Ser116 in NPCs that received 100 μM TBHP treatment. The mean ± standard deviation (SD) is shown by the error bars. NS: not significant.

Based on the analysis of degenerative NP and control NP tissues in the dataset GSE67566, a heat map showing the elevated expression of various circRNAs in degenerated NP tissues was generated (Figure S1A). We screened the circRNAs involved in regulating cellular ROS injury through literature research and ultimately chose circFOXO3 for our study [21,22,23,24,25,26,27]. To further explore circFOXO3 expression, we performed qPCR on NP tissues from 10 IVDD patients and 10 controls. The findings confirmed significantly higher expression levels of circFOXO3 in the IVDD patients in contrast to the normal group (Figure 1A). To validate the circular nature of circFOXO3, we amplified cDNA using divergent and convergent primers. The PCR product band of linear FOXO3 mRNA pretreated with Ribonuclease R (RNase R) disappeared in cDNA amplified by convergent primers, indicating the circular structure of circFOXO3 (Figure S1B). The head-to-tail splicing location of circFOXO3 was verified using Sanger sequencing (Figure S1C, arrow). In vitro, treatment of NPCs with the ROS inducer *tert*-butyl hydroperoxide (TBHP) at different concentrations and time points indicated that circFOXO3 expression was significantly upregulated more than 2-fold after 24 h of 100 μM TBHP treatment (Figure 1B; Figure S1D). Meanwhile, under these conditions, the intracellular ROS level in NPCs was significantly increased (Figure S1E,F). Comparatively, treatment with 150 μM TBHP resulted in higher circFOXO3 upregulation, and the condition of NPCs after this treatment was not suitable for subsequent experimental detection. Consequently, we selected the treatment conditions of 24 h and 100 μM TBHP for subsequent experiments.

### 3.2. TBHP Treatment Activates Oxeiptosis-Related Pathway in NPCs In Vitro

Previous studies have shown that exogenous ROS can induce apoptosis, necrosis, autophagy, and ferroptosis in NPCs [30,31,32]. To investigate the involvement of these pathways induced in NPCs by TBHP treatment, we utilized various inhibitors, including Z-VAD (a caspase-dependent apoptosis inhibitor), Necrostatin-1 (Nec-1; a necrosis inhibitor), 3-Methyladenine (3-MA; an autophagy inhibitor), Ferrostatin-1 (Fer-1; a ferroptosis inhibitor), and N-Acetyl-L-cysteine (NAC; an ROS inhibitor). The CCK-8 assay was utilized to examine the cell viability of NPCs. The results revealed that except NAC, the inhibitors (Z-VAD, Nec-1, 3-MA, and Fer-1) were unable to reverse the decline in NPC viability (Figure S2A), suggesting that the TBHP-induced cell death in NPCs was likely mediated by a mechanism independent of apoptosis, necroptosis, autophagy, and ferroptosis.

To investigate whether TBHP induces oxeiptosis in NPCs, we conducted immunofluorescence experiments using COX IV as a specific mitochondria marker, co-localizing it with KEAP1 and PGAM5. After TBHP treatment, we observed that KEAP1 lost its co-localization with COX IV, while PGAM5 maintained its co-localization with COX IV (Figure S2B), which is one of the important manifestations when oxeiptosis is activated [7,15]. Next, we assessed the mRNA expression of KEAP1 and PGAM5 in NPCs subjected to treatment with TBHP at different concentrations (Figure S2C,D). Additionally, we performed TEM imaging and conducted Western blot analysis. The TEM images displayed features associated with oxeiptosis, like enlargement of the nucleus and membrane blebbing (Figure S2E) [7,14]. Western blot analysis indicated changes in protein expression consistent with the activation of the oxeiptosis-related pathway. Specifically, the expression of KEAP1 and PGAM5 increased, while the ratio of phosphorylation of AIFM1 at Ser116 (p-AIFM1)/AIFM1 decreased (Figure 1C–G). Furthermore, knocking down KEAP1 or PGAM5 could attenuate the decrease in NPC viability caused by TBHP (Figure S2F–J). Taken together, our findings powerfully indicate that TBHP causes oxeiptosis in NPCs in vitro.

### 3.3. Knocking down circFOXO3 Suppresses Oxidative Stress-Induced Oxeiptosis in NPCs

**Figure 2:**
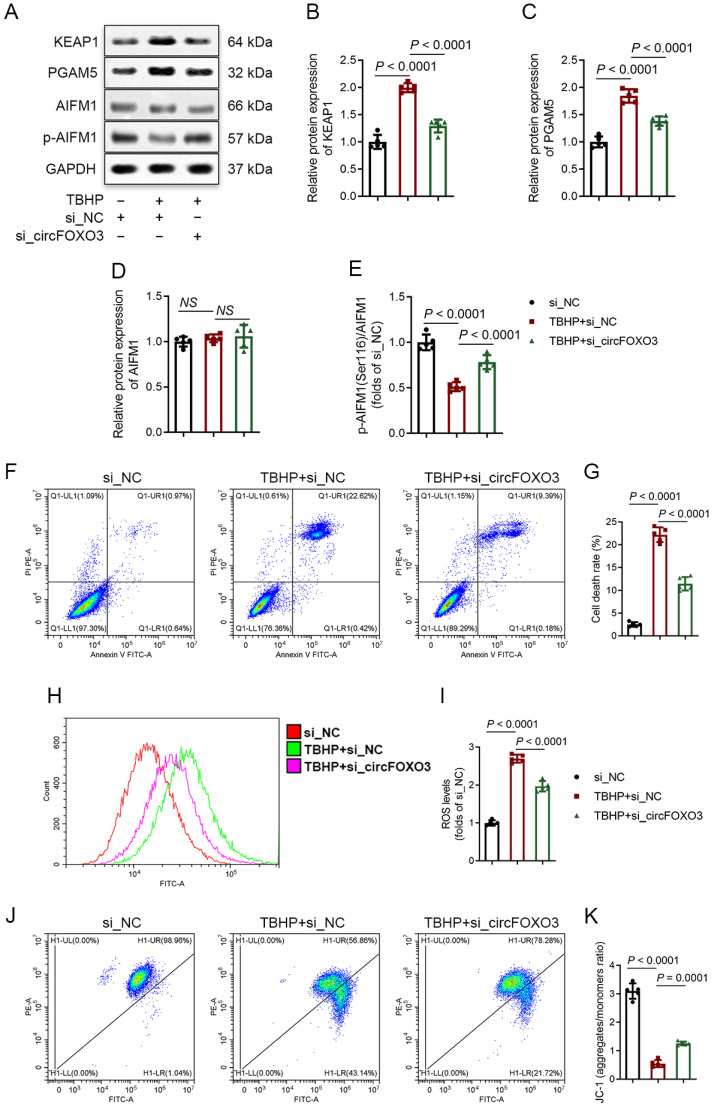
Knockdown of circFOXO3 suppresses oxidative stress-induced oxeiptosis in NPCs. (**A**–**E**) Western blot analysis of protein expression of KEAP1, PGAM5, and AIFM1 phosphorylated at Ser116 in NPCs treated with TBHP and transfected with NC small interfering RNA (siRNA) or circFOXO3 siRNA. (**F**–**K**) Flow cytometry analysis of Annexin V/Propidium Iodide (PI) (**F**,**G**), DCFH-DA (**H**,**I**), and JC-1 (**J**,**K**). The mean ± SD is shown by the error bars. NC: negative control. NS: not significant.

We established a specific small interfering RNA (siRNA) to successfully knock down circFOXO3 expression (Figure S3A–C) to investigate the function of circFOXO3 in NPCs. Concurrently, we constructed an adenovirus overexpressing circFOXO3 (Figure S3D–F). Through Western blot analysis, we observed that knocking down circFOXO3 partially reversed the TBHP-induced upregulation of KEAP1 and PGAM5 proteins and attenuated the dephosphorylation of p-AIFM1 (Figure 2A–E). Furthermore, flow cytometry analysis was performed to assess cell death rates, ROS levels, and changes in MMP in NPCs. The results demonstrated that circFOXO3 knockdown attenuated TBHP-induced cell death and TBHP-increased ROS levels and stabilized MMP (Figure 2F–K). These findings were consistent with Mitotracker Red staining, which also showed changes in MMP (Figure S3G,H).

To confirm that knocking down circFOXO3 protects NPCs by inhibiting oxeiptosis, we conducted co-transfections of KEAP1 siRNA or PGAM5 siRNA with circFOXO3 siRNA, and relevant analyses were performed. The Western blot results revealed no significant differences between individual transfections of KEAP1 siRNA or PGAM5 siRNA and co-transfection with circFOXO3 siRNA in terms of inhibiting the dephosphorylation of p-AIFM1 (Figure S4A–E). Similarly, the flow cytometry results showed no significant variations between individual transfections and co-transfection in terms of inhibiting NPC death, decreasing ROS levels, and stabilizing MMP (Figure S4F–K), indicating that the knockdown of circFOXO3 effectively attenuates the oxeiptosis in NPCs.

### 3.4. CircFOXO3 Functions as a Molecular Sponge to Target and Downregulate miR-185-3p and miR-939-5p in NPCs

**Figure 3:**
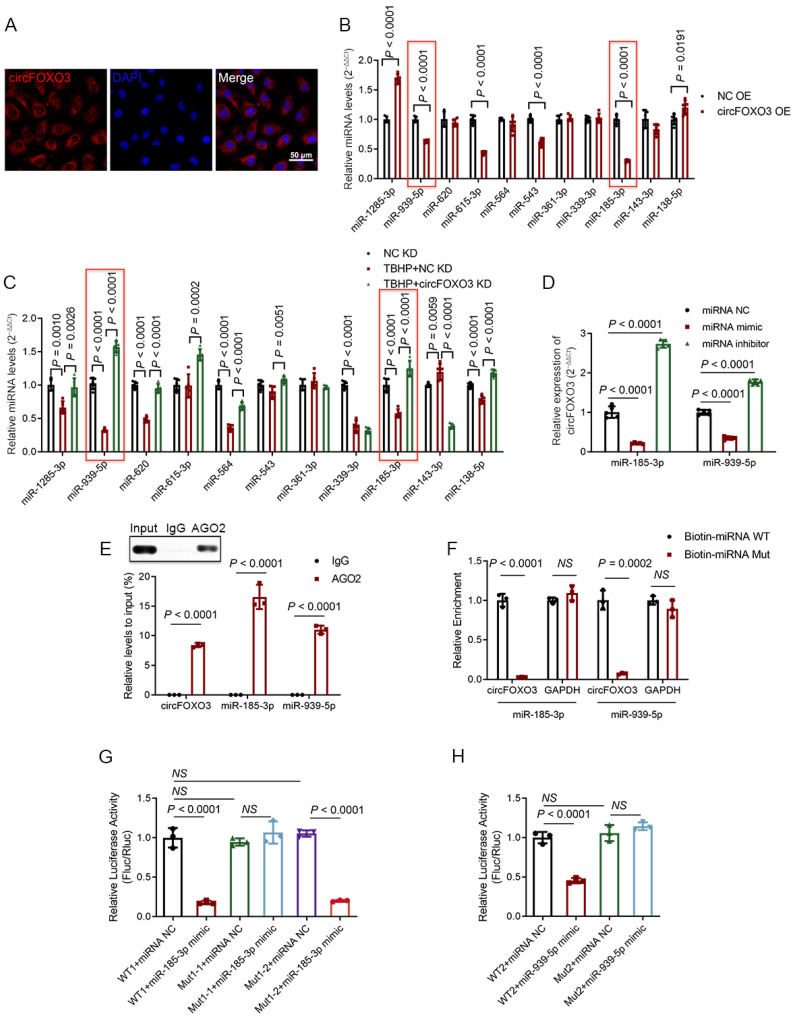
CircFOXO3 functions as a sponge for miR-185-3p and miR-939-5p. (**A**) RNA Fluorescence In Situ Hybridization (RNA FISH) showing the expression of circFOXO3 (red) in NPCs. Nuclei were stained with 4′,6-diamidino-2-phenylindole (DAPI) (blue). Scale bar = 50 μm. (**B**,**C**) Expression levels of miR-185-3p and miR-939-5p (red frame) decreased upon circFOXO3 overexpression (**B**) and treatment with TBHP (**C**) and increased after circFOXO3 knockdown (**C**). (**D**) Expression of circFOXO3 decreased upon ectopic transfection of miR-185-3p or miR-939-5p mimics and increased after inhibition of miR-185-3p or miR-939-5p. (**E**) RNA Immunoprecipitation (RIP) assays were performed using an anti-Argonaute-2 (AGO2) or anti-Immunoglobulin G (IgG) antibody in NPC lysate. (**F**) Biotinylated WT/Mut miR-185-3p or miR-939-5p was transfected into NPCs, and qPCR was used to detect circFOXO3 enrichment. (**G**,**H**) Luciferase reporter assays in HEK293T cells were performed with WT/Mut circFOXO3 luciferase reporter plasmids and microRNA (miRNA) mimics. Site 1-2 was validated as a non-functional target (**G**). The mean ± SD is shown by the error bars. WT: wild type. Mut: mutant. NC: negative control. OE: overexpression. KD: knockdown. NS: not significant.

As previously reported, circFOXO3 is predominantly located in the cytoplasm of various cell types [21,23,25,33,34]. To understand the subcellular localization of circFOXO3 in human NPCs, we conducted RNA FISH experiments, and the results demonstrated that circFOXO3 is mainly located in the cytoplasm of human NPCs (Figure 3A), which may be related to the potential function of circFOXO3 as an miRNA molecular sponge.

To investigate the miRNA molecular sponge function of circFOXO3, we performed a comprehensive search and analysis of miRNA targets using various online public databases. We also considered the predicted matching sequences from the database and information available in previous literature reports [35,36,37,38,39,40,41,42,43,44,45] and finally selected 11 potential downstream miRNAs for further detection (Figure S5A). Through knockdown and overexpression of circFOXO3, we observed corresponding changes in the miR-185-3p and miR-939-5p expression levels, showing upregulation and downregulation, respectively (Figure 3B,C). Furthermore, when we transfected NPCs with miR-185-3p or miR-939-5p mimic, the expression level of circFOXO3 was found to decrease. Conversely, when we inhibited miR-185-3p or miR-939-5p, the expression level of circFOXO3 increased (Figure 3D).

To verify the direct binding of circFOXO3 to miR-185-3p and miR-939-5p, we conducted anti-AGO2 RIP experiments. The results demonstrated that the anti-AGO2 group enriched circFOXO3 and miR-185-3p/miR-939-5p, while the IgG group did not show similar enrichment (Figure 3E). To further elucidate the specific matching sequences between circFOXO3 and miR-185-3p/miR-939-5p, we performed dual-luciferase reporter gene and RNA pull-down assays. In the RNA pull-down assay, the results demonstrated that the wild-type miR-185-3p/miR-939-5p showed a greater enrichment of circFOXO3 compared to the mutant (Figure 3F; Figure S5B). For the dual-luciferase reporter gene assay, the results indicated that the wild-type plasmid significantly reduced luciferase activity compared to the mutant plasmid (Figure 3G,H; Figure S5C).

### 3.5. MiR-185-3p and miR-939-5p Are Downregulated in Degenerative NP Tissues and Participate in the Regulation of Oxeiptosis

**Figure 4:**
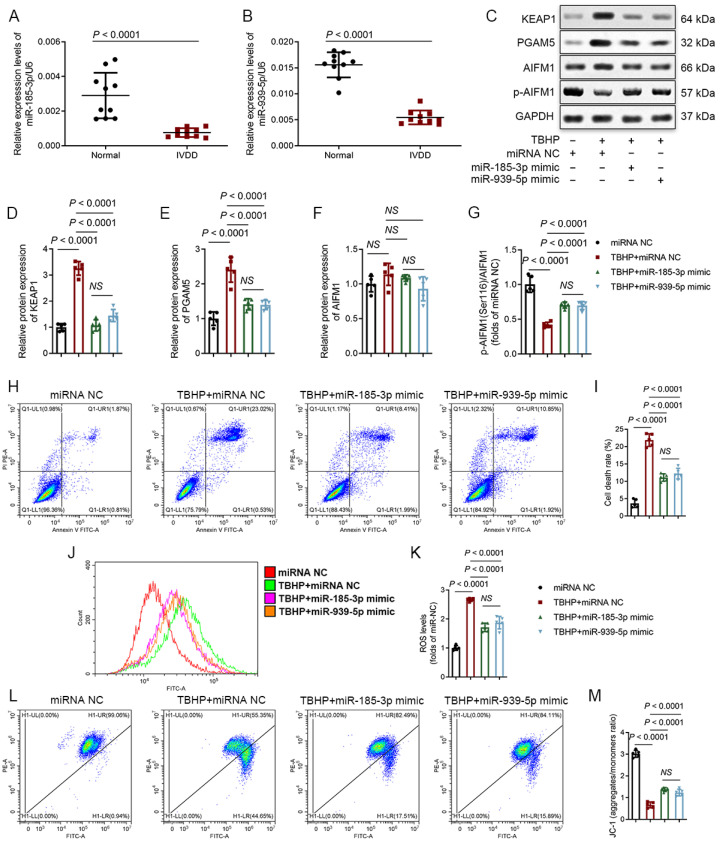
MiR-185-3p and miR-939-5p suppress oxidative stress-induced oxeiptosis in NPCs. (**A**,**B**) The relative expression levels of miR-185-3p (**A**) and miR-939-5p (**B**) were significantly decreased in IVDD NP tissues compared to normal NP tissues (IVDD vs. normal controls: *n* = 10, respevtively). (**C**–**G**) Western blot analysis showing the protein expression levels of KEAP1, PGAM5, and AIFM1 phosphorylated at Ser116 in NPCs treated with TBHP and transfected with miRNA NC, miR-185-3p mimic, or miR-939-5p mimic. (**H**–**M**) Flow cytometry analysis of Annexin V/PI (**H**,**I**), DCFH-DA (**J**,**K**), and JC-1 (**L**,**M**). The mean ± SD is shown by the error bars. NC: negative control. NS: not significant.

Next, we investigated the expression levels of miR-185-3p and miR-939-5p in NP tissues and found that miR-185-3p and miR-939-5p were downregulated in the IVDD group (Figure 4A,B). To further understand their functions, we treated NPCs that were subjected to miR-185-3p/miR-939-5p mimic transfection. The results showed that transfection of miR-185-3p/miR-939-5p mimics weakened the activation of the KEAP1-PGAM5-AIFM1 pathway (Figure 4C–G). Additionally, flow cytometry analysis exhibited that transfection of miR-185-3p/miR-939-5p mimics attenuated TBHP-induced NPC death and TBHP-increased ROS levels and stabilized MMP (Figure 4H–M). These functional effects were consistent with the results observed with circFOXO3 knocked down. However, no significant variations were detected between the influences of miR-185-3p and miR-939-5p in these experiments.

### 3.6. ASIC1 Is a Common Target of miR-185-3p and miR-939-5p

**Figure 5:**
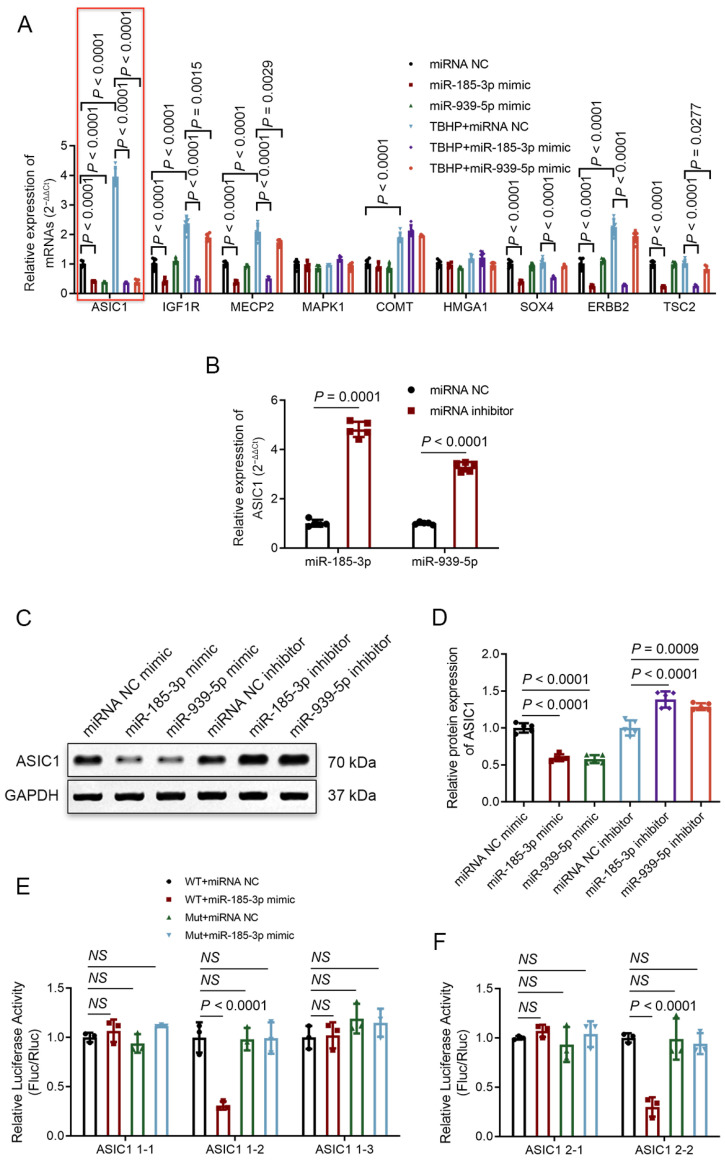
Acid-sensing ion channel subunit 1 (ASIC1) is a common endogenous target of miR-185-3p and miR-939-5p. (**A**,**B**) Quantitative PCR analysis showing the mRNA expression levels of ASIC1 (red frame), which decreased upon miR-185-3p or miR-939-5p overexpression (**A**) and increased after treatment with TBHP (**A**) and/or knockdown of miR-185-3p or miR-939-5p (**B**). (**C**,**D**) Western blot analysis of ASIC1 protein expression levels in NPCs. (**E**,**F**) Luciferase reporter assays in HEK293T cells were performed with WT/Mut ASIC1 luciferase reporter plasmids and miRNA mimics. ASIC1 1-1, 1-3 (**E**), and 2-1 (**F**) sites were validated as non-functional targets. The mean ± SD is shown by the error bars. WT: wild type. Mut: mutant. NC: negative control. NS: not significant.

By searching the TargetScan online database and referring to previous studies [46,47,48,49,50,51,52,53,54,55], we observed that the ASIC1 mRNA and protein expression levels in NPCs were downregulated or upregulated when miR-185-3p or miR-939-5p was overexpressed or knocked down (Figure 5A–D). To verify the direct targeting regulation between miR-185-3p/miR-939-5p and ASIC1, we mutated the potential miRNA binding site sequence in the 3′UTR region of ASIC1 and introduced it into a luciferase reporter plasmid. The plasmid was then co-transfected with miR-185-3p/miR-939-5p mimics, and the results demonstrated that transfection of the wild-type plasmid significantly decreased luciferase activity in contrast to transfection with the mutant plasmid (Figure 5E,F; Figure S5D), which further clarified the direct interaction between miR-185-3p/miR-939-5p and ASIC1.

### 3.7. Degenerative NP Tissues Express a Higher Level of ASIC1, and circFOXO3 Intervenes with Oxeiptosis in NPCs by Regulating ASIC1 through Sponge miR-185-3p and miR-939-5p

**Figure 6:**
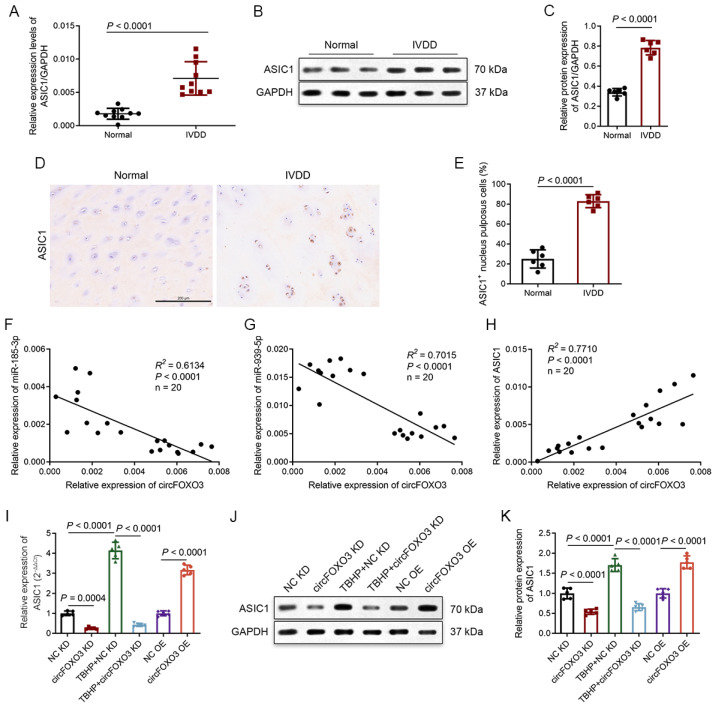
CircFOXO3 modulates the expression of ASIC1 by targeting miR-185-3p and miR-939-5p. (**A**) ASIC1 mRNA transcript levels were significantly increased in IVDD NP tissues compared to normal NP tissues (IVDD vs. normal controls: *n* = 10, respectively). (**B**,**C**) Western blot analysis of ASIC1 protein was conducted in IVDD NP tissues (*n* = 6, respectively) and normal NP tissues (*n* = 6, respectively). Representative expression patterns are presented (**B**). (**D**,**E**) Representative immunohistochemistry (IHC) staining of IVDD NP tissues and normal NP tissues. Scale bar = 200 μm (**D**). Quantification of ASIC1-immunopositive NPCs in IVDD NP tissues and normal NP tissues. Data are presented as a percentage of positive cells (**E**). (**F**–**H**) CircFOXO3 expression was inversely correlated with the expression of miR-185-3p (**F**) and miR-939-5p (**G**) and positively correlated with the mRNA expression of ASIC1 (**H**) in NPCs. (**I**–**K**) The relative ASIC1 mRNA and protein expression levels in NPCs were analyzed by qPCR (**I**) and Western blot assay (**J**,**K**). The mean ± SD is shown by the error bars. OE: overexpression. KD: knockdown.

Several studies have reported that the expression levels of ASIC1 in lumbar NP tissues are significantly increased in the IVDD group [46,47]. Consistent with earlier studies, our results showed elevated expression of ASIC1 in the IVDD group compared to normal controls (Figure 6A–E). We further conducted correlation analyses on the RNA expression of circFOXO3, miR-185-3p, miR-939-5p, and ASIC1 in both IVDD patients and normal controls. The results revealed that circFOXO3 and miR-185-3p/miR-939-5p were negatively correlated (Figure 6F,G), while circFOXO3 exhibited a positive correlation with ASIC1 (Figure 6H). To investigate the regulatory relationship between circFOXO3 and ASIC1, we modulated the expression of circFOXO3 in NPCs. The results revealed that the upregulation and downregulation of circFOXO3 were associated with corresponding changes in the expression of ASIC1 (Figure 6I–K).

To confirm the regulatory role of circFOXO3 as a molecular sponge, influencing the expression of ASIC1 by targeting miR-185-3p and miR-939-5p, and its involvement in the oxeiptosis process of NPCs, we conducted rescue experiments. The results of qPCR, Western blot, and CCK-8 assays showed that after TBHP treatment, co-transfection of circFOXO3 siRNA with an miR-185-3p inhibitor and/or miR-939-5p inhibitor effectively reversed the impact of circFOXO3 siRNA transfection alone (Figure S6A–K).

### 3.8. CircFOXO3 Affects the Susceptibility of NPCs to TBHP-Induced Oxeiptosis by Regulating the Expression of ASIC1

**Figure 7:**
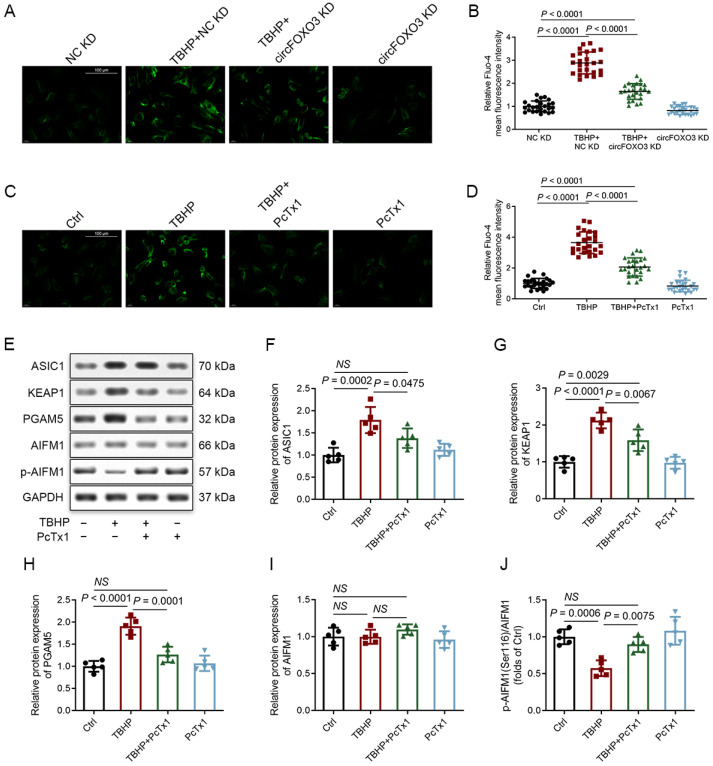
ASIC1 mediates oxeiptosis by triggering Calcium ion (Ca^2+^) influx. (**A**–**D**) Ca^2+^ levels in NPCs were detected using Fluo-4 (green). Scale bar = 100 μm (**A**,**C**). The mean fluorescence intensity of Fluo-4 was calculated using ImageJ software (data from 100 cells from 3 individual experiments), and the fluorescence intensity of the NC KD or control group cells was set as 1 for normalization (**B**,**D**). (**E**–**J**) Western blot analysis of protein expression of ASIC1, KEAP1, PGAM5, and AIFM1 phosphorylated at Ser116 in NPCs after treatment for 30 min with 20 nM Psalmotoxin 1 (PcTx1) (#ab120483; Abcam, Cambridge, UK) followed by 24 h of treatment with H_2_O (control) or 100 μM TBHP. The mean ± SD is shown by the error bars. NC: negative control. KD: knockdown. NS: not significant.

ASIC is a type of cation channel located on the cell membrane. It opens in response to the binding of protons, resulting in the influx of extracellular cations and triggering various cellular activities [56,57,58,59]. Unlike other ASIC subunits, ASIC1 is unique in mediating the influx not only of Na^+^ but also of extracellular Ca^2+^ [58,60]. Given the functional significance of ASIC1, we conducted Fluo-4 staining to measure the levels of intracellular Ca^2+^ in NPCs. Following TBHP treatment, we observed increased Ca^2+^ levels in NPCs, which could be mitigated by either knocking down circFOXO3 or using the ASIC1-specific inhibitor Psalmotoxin 1 (PcTx1) (Figure 7A–D). Importantly, PcTx1 not only decreased the expression of ASIC1 but also suppressed the activation of the KEAP1-PGAM5-AIFM1 pathway induced by TBHP (Figure 7E–J).

However, when circFOXO3 was overexpressed alone, it resulted in lower Ca^2+^ levels compared to the levels induced by TBHP alone, and co-expression of circFOXO3 with TBHP further increased the Ca^2+^ levels in NPCs (Figure S7A,B). The analysis of CCK-8, qPCR, and Western blot data revealed that overexpression of circFOXO3 alone was not sufficient to completely replicate the changes in cell viability and activation of the KEAP1-PGAM5-AIFM1 pathway by TBHP alone, while co-expression of circFOXO3 with TBHP further enhanced these effects (Figure S7C–K). These results indicate that circFOXO3 influences the susceptibility of NPCs to TBHP-induced oxeiptosis by regulating downstream ASIC1 expression rather than causing oxeiptosis independently, and the Ca^2+^ influx triggered by ASIC1 is implicated in oxeiptosis in NPCs.

### 3.9. Inhibition of circFOXO3 Alleviates IVDD In Vivo

To verify the mechanism and function of circFOXO3, we established a mouse IVDD model through AF puncture and locally injecting an AAV containing plasmids with fLuc or enhanced green fluorescent protein tags attached to the plasmid. The in vivo monitoring of IVD-targeted ability was performed utilizing the live imaging of the mice, and fluorescence imaging was used to verify the efficacy of the in vivo delivery of AAV into the disc (Figure S8A,B).

**Figure 8:**
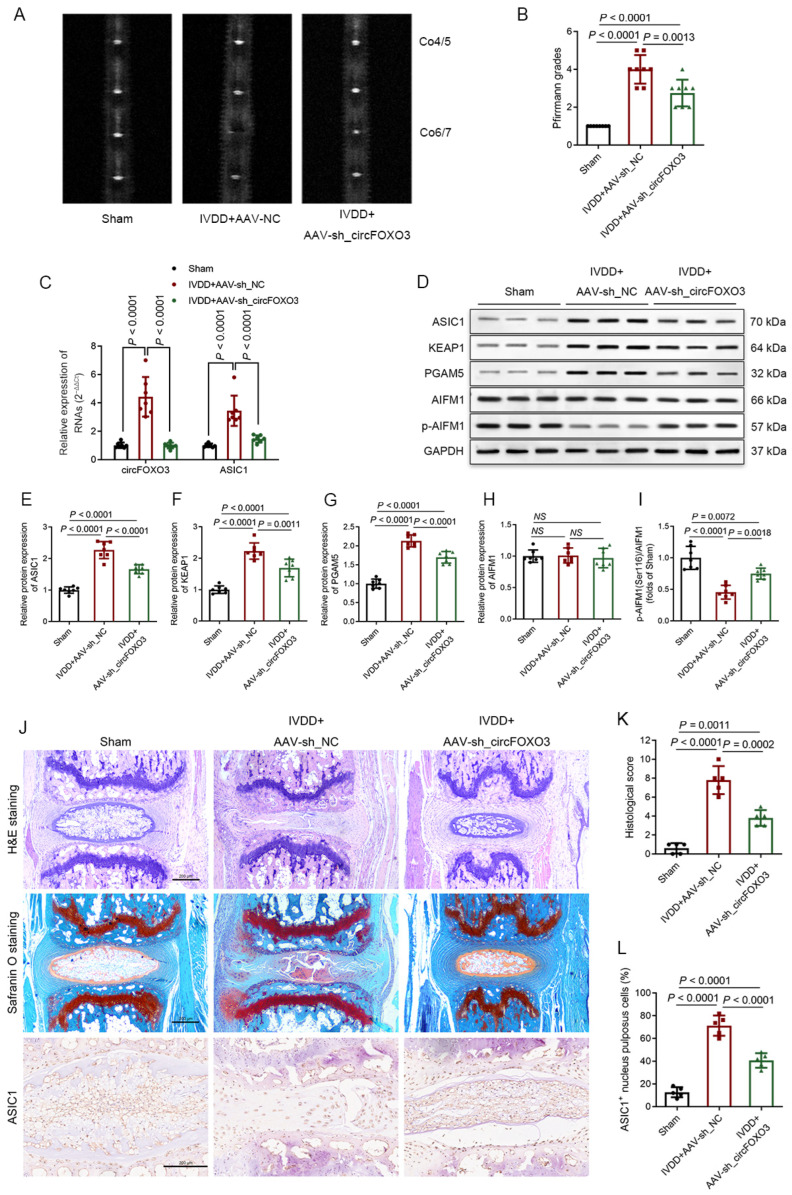
Silencing circFOXO3 in vivo attenuated IVDD development. (**A**,**B**) IVDD was evaluated by magnetic resonance imaging (MRI) analysis. A significant rise in Pfirrmann grading was detected at 4 weeks after surgery in mice that received adeno-associated virus (AAV)-sh_circFOXO3 treatment. (**C**) Quantitative PCR showed that compared with the sham group, the IVDD+AAV-sh_NC group expressed higher levels of circFOXO3 and ASIC1 RNA, and the IVDD+AAV-sh_circFOXO3 group showed a significant decrease in circFOXO3 and ASIC1 RNA. (**D**–**I**) Western blot analysis showed that knocking down circFOXO3 attenuated surgery-induced activation of the oxeiptosis-related pathway and upregulation of ASIC1. (**J**–**L**) H&E and Safranin O-Fast Green staining evaluated the histological changes of the annulus fibrosus (AF)-punctured intervertebral discs. Scale bar = 200 μm (**J**). Histological score showing a significant decrease in mice that received AAV-sh_circFOXO3 treatment (**K**). Representative IHC staining of the AF punctured intervertebral discs. Scale bar = 200 μm (**J**). Quantification of ASIC1-immunopositive NPCs in the intervertebral discs of each group. Data are presented as percentages of positive cells (**L**). The mean ± SD is shown by the error bars. NC: negative control. NS: not significant.

Interestingly, circFOXO3 knockdown could alleviate IVDD in vivo. MRI analysis showed degeneration of IVDs between mice in different treatment groups, and the circFOXO3 knockdown group was found to have a lower Pfirrmann grading than the NC group (Figure 8A,B). Quantitative PCR and Western blot analysis exhibited that knocking down circFOXO3 diminishes ASIC1 expression levels and weakens the activation of the KEAP1-PGAM5-AIFM1 pathway in NP tissues in vivo (Figure 8C–I). Western blot analysis also showed that extracellular matrix degradation could be effectively prevented by knocking down circFOXO3 (Figure S8C–G). Histological evaluation and IHC detection of the AF-punctured IVDs showed that knocking down circFOXO3 significantly attenuates both cellular and structural degeneration of NP and AF and reduces the proportion of ASIC1-immunopositive NPCs (Figure 8J–L). TUNEL staining also demonstrated a reduced proportion of TUNEL-positive NPCs in the circFOXO3 knockdown group (Figure S8H,I), which indicated a reduced cell death rate.

## 4. Discussion

In recent years, several studies have identified various circRNAs related to the development of IVDD that function by targeting different miRNAs or RNA-binding proteins [16,17,18,19,20], thereby regulating diverse cellular activities. Previous research has shown that circFOXO3 is upregulated in different tissues and cells in response to exogenous ROS, and silencing circFOXO3 has been found to counteract the detrimental effects of ROS [21,22,23,24,25,26,27]. This study is the first to explore the functions and underlying mechanisms of circFOXO3 in ROS injury and oxeiptosis in NPCs. CircFOXO3 was found to be upregulated in NPCs in degenerative IVD and in NPCs exposed to in vitro administration of TBHP (exogenous ROS), which modulates the expression of ASIC1 through the sponging effect on miR-185-3p and miR-939-5p. As a result, we have established the presence of the circFOXO3-miR-185-3p/miR-939-5p-ASIC1 axis in NPCs. This axis enhances the susceptibility of NPCs to ROS-induced oxeiptosis, consequently accelerating the progression of IVDD. Inhibition of circFOXO3 was shown to mitigate TBHP-induced oxeiptosis, and in vivo, silencing of circFOXO3 was found to delay the IVDD progression in a mouse model. Consequently, circFOXO3 is one of the factors contributing to the progression of IVDD by mediating oxeiptosis in NPCs.

MiR-185-3p and miR-939-5p have been revealed to play essential roles in regulating the occurrence and development of different diseases [61,62,63,64]. In this study, we provide the first description of the function and mechanism of miR-185-3p and miR-939-5p in IVDD, uncovering a shared downstream target gene, ASIC1. During IVDD, miR-185-3p and miR-939-5p are downregulated in NPCs, leading to the upregulation of ASIC1. We observed similar changes in NPCs undergoing oxeiptosis induced by TBHP in vitro. By using an miR-185-3p/miR-939-5p mimic and the ASIC1-specific inhibitor PcTx1, we could effectively inhibit oxeiptosis. These findings are consistent with previous studies showing elevated expression of ASIC1 in degenerative IVDs, where the specific inhibition of ASIC1 promotes the survival of NPCs in the presence of exogenous stimuli [46,47].

Previous studies on ASIC1 and ROS have primarily focused on the proton-gated characteristics of ASIC1, often involving stimulation with acidic external environments. For instance, Zai et al. conducted research on rat chondrocytes and found that extracellular acidification leads to an elevation in mitochondrial ROS levels by activating ASIC1 [65]. In another study, Zhao et al. reported that extracellular lactate regulated intercellular ROS levels through ASIC1 and ASIC3 in NP cells [47]. In research performed by Wu et al. in 2021, to clarify the influence of ROS on ASIC1, it was reported that exogenous H_2_O_2_ could elevate the expression of ASIC1 through the JNK pathway, leading to enhanced ASIC currents in neuronal cell lines (NS20Y) [66]. In our present study, we observed that both TBHP treatment and overexpression of circFOXO3 resulted in an upregulation of ASIC1 expression. However, it was found that overexpression of circFOXO3 alone did not activate the oxeiptosis-related pathway to the same extent as TBHP treatment, while co-expression of circFOXO3 with TBHP further activated the oxeiptosis-related pathway compared to TBHP treatment alone. This evidence suggests that the proton-gated characteristics of ASIC1 play an important role in oxeiptosis in NPCs.

ASIC1 is the only subtype among the ASICs permeable to Ca^2+^. Previous research has linked ASIC1 to calcium-dependent cell death in degenerated IVDs [67,68]. Our study further revealed that the accumulation of Ca^2+^ in NPCs with TBHP treatment was higher compared to that in NPCs with circFOXO3 overexpression alone, and this could be related to the impact of exogenous ROS and the opening degree of ASIC1. Notably, pretreatment with PcTx1, an ASIC1-specific inhibitor, reduced the accumulation of Ca^2+^ and the activation of the oxeiptosis-related pathway in NPCs following TBHP treatment, suggesting that the occurrence of oxeiptosis could be dependent on the opening of ASIC1 and the subsequent influx of Ca^2+^. Within this mechanism, circFOXO3 plays a role in modulating the potential influx of Ca^2+^ in NPCs by regulating the expression of ASIC1. This alteration in ASIC1 expression consequently modifies the susceptibility of NPCs to oxeiptosis, thereby influencing the progression of IVDD.

Previous studies have highlighted the intricate relationship between Ca^2+^ and ROS [69,70,71]. Herein, we also observed a correlation between these factors as the silencing of circFOXO3 and transfection of miR-185-3p and miR-939-5p mimics were found to reverse the increase in ROS levels and the changes in MMP in NPCs after TBHP treatment. Similarly, as previously mentioned, the inhibition of ASIC1 has been shown in multiple studies to effectively suppress the elevation of ROS levels and the changes in MMP in various cells, including NPCs, so we did not repeat it in our experiments. Nevertheless, our results did not allow us to determine whether Ca^2+^ regulates oxeiptosis by affecting intracellular ROS levels or whether there are other underlying mechanisms, urging the need for further experimental evidence.

This study reports that oxeiptosis in NPCs is one of the mechanisms underlying the progression of IVDD, and inhibiting the accumulation and damage of ROS is a feasible strategy to delay the progression of IVDD. Although molecular therapeutic approaches for IVDD are still in the exploratory stage, targeted detection of circFOXO3 or oxeiptosis may help clinicians assess the progression status and treatment prognosis of IVDD.

In conclusion, our study provides evidence supporting the presence of oxeiptosis in NPCs. We have indicated that circFOXO3 upregulates the expression of ASIC1 by targeting miR-185-3p and miR-939-5p, which enhances the susceptibility of NPCs to ROS-induced oxeiptosis and promotes the progression of IVDD. Consequently, this leads to a greater loss of NPCs within IVDs among individuals with elevated circFOXO3 expression under the same adverse stimulus. In summary, the upregulation of circFOXO3 promoting oxeiptosis in NPCs is one of the molecular factors contributing to the progression of IVDD.

## Data Availability

All data are available from the corresponding authors upon reasonable request. CircRNA expression data were downloaded from Gene Expression Omnibus, GEO (https://www.ncbi.nlm.nih.gov/geo/, accessed on 24 March 2022).

## Supplementary Materials

The following supporting information can be downloaded at https://www.mdpi.com/article/10.3390/biomedicines12030678/s1, Figure S1: Characteristics and expression of circFOXO3 in nucleus pulposus cells (NPCs). Figure S2: TBHP induces oxeiptosis in NPCs. Figure S3: The interference efficiency of circFOXO3 expression in NPCs. Figure S4: Knockdown of KEAP1 and PGAM5 counteracts the protective effect of circFOXO3 knockdown on oxeiptosis in NPCs. Figure S5: MiRNA screening and targeted sequence information. Figure S6: The rescue experiment confirmed the existence of the circFOXO3-miR-185-3p/miR-939-5p-ASIC1 axis in NPCs. Figure S7: Overexpression of circFOXO3 increases the susceptibility of NPCs to oxeiptosis. Figure S8: Additional in vivo experimental results. Table S1: Primers list for qPCR. Table S2: Sequences of siRNAs. Table S3: Sequences of mimic and inhibitor of miRNAs. Table S4: Sequences of RNA FISH probe and AAV-sh_circFOXO3.
