# Supplementary material for: Circular RNA CircFOXO3 Functions as a Competitive Endogenous RNA for Acid-Sensing Ion Channel Subunit 1 Mediating Oxeiptosis in Nucleus Pulposus
Source: Biomedicines. 2024 Mar 18;12(3):678. doi: 10.3390/biomedicines12030678 (PMC10968533; doi:10.3390/biomedicines12030678)
Supplement: Supplementary file 1 [file biomedicines-12-00678-s001.zip › biomedicines-2895848-supplementary.pdf]

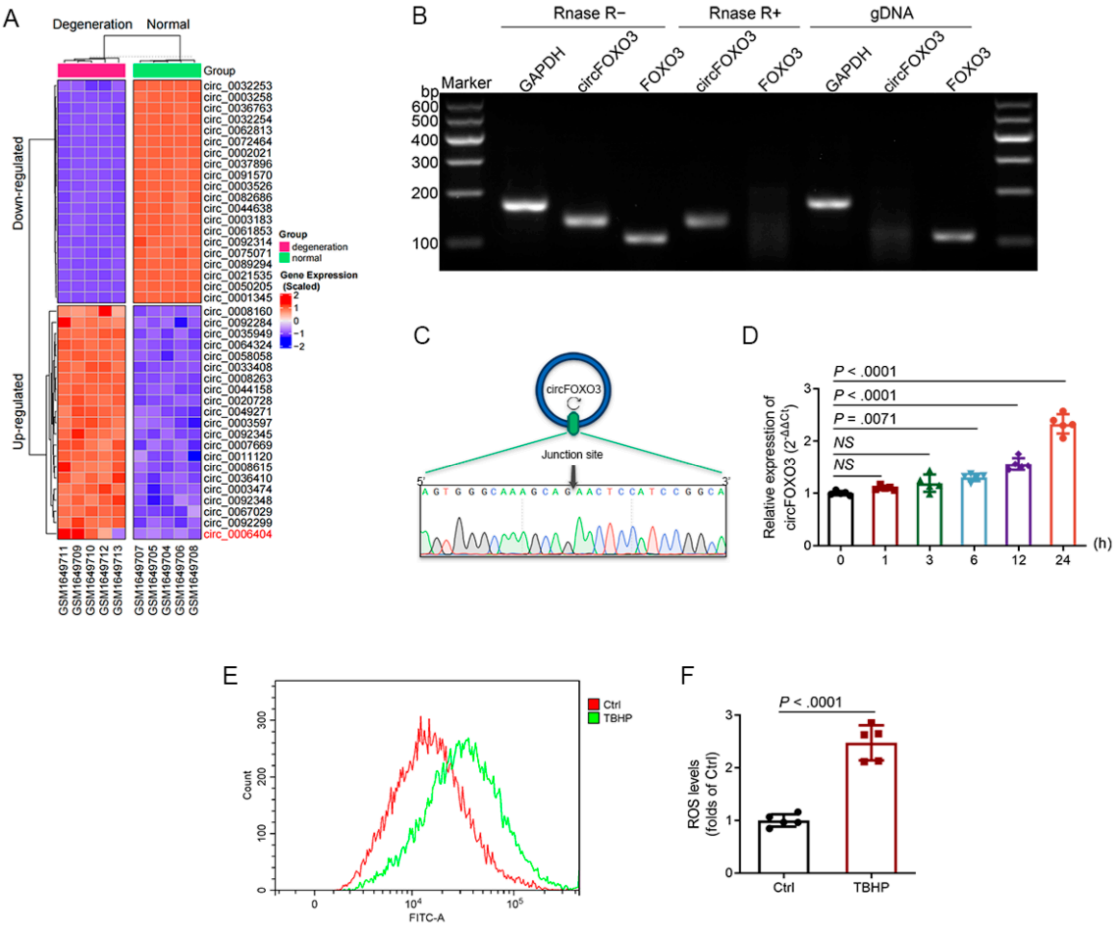

Supplementary Figure S1. Characteristics and expression of circFOXO3 in nucleus pulposus cells (NPCs)

(A) Hierarchical cluster analysis of circular RNAs (circRNAs) differentially expressed between intervertebral disc degeneration (IVDD) and normal nucleus pulposus (NP) tissues, based on data from the Gene Expression Omnibus (GEO) database (GSE67566). Each column represents a sample, and each row represents a circRNA. The red strip indicates high relative expression, while the blue strip indicates low relative expression. (B) PCR analysis of total RNA from NPCs with or without Ribonuclease R (RNase R) treatment, confirming the circular nature of circFOXO3. (C) Sanger sequencing validation of circFOXO3 using PCR with specific divergent flanking primers. The black arrow exhibits the "head-to-tail" splicing junction of circFOXO3. (D) Concentration-course analysis of circFOXO3 relative expression in NPCs treatment with TBHP. (E, F) Flow cytometry analysis of 2′,7′-Dichlorodihydrofluorescein diacetate (DCFH-DA) showed intracellular ROS levels of NPCs treated by TBHP and control. The mean ± SD is shown by the error bars. NS: not significant.

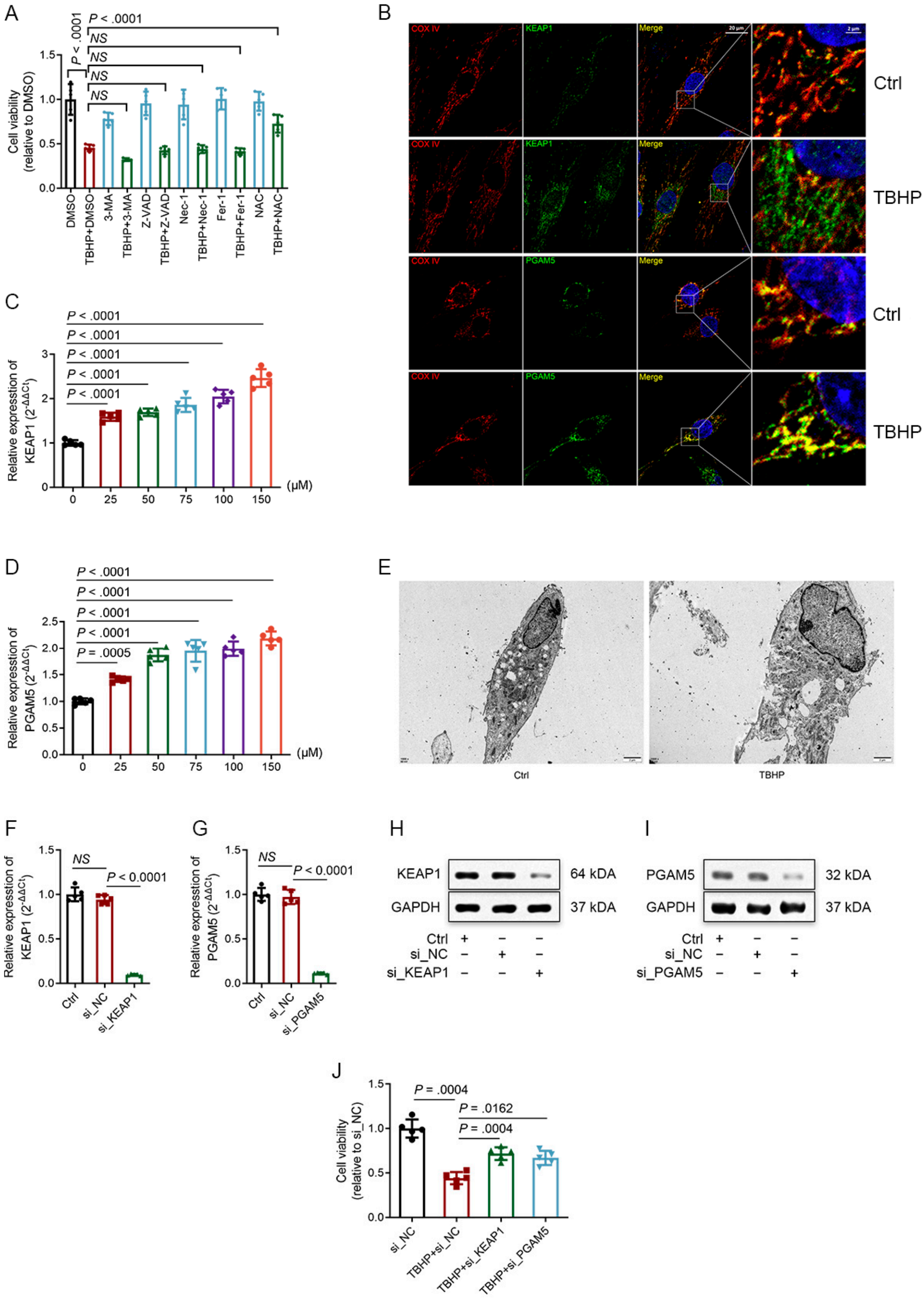

Supplementary Figure S2. TBHP induces oxeiptosis in NPCs

(A) Viability of NPCs assessed using cell counting kit-8 (CCK-8) assays after treatment for 1 h with DMSO (control), 20 μM Z-VAD (a caspase-dependent apoptosis inhibitor; #HY-16658B, MCE), 20 μM Necrostatin-1

(Nec-1) (a necrosis inhibitor; #HY-15760, MCE), 20 μM Ferrostatin-1 (Fer-1) (a ferroptosis inhibitor; #HY-100579, MCE), or 2 h with 10 mM 3-Methyladenine (3-MA) (an autophagy inhibitor; #HY-19312, MCE), or 30 min with 10 mM N-Acetyl-L-cysteine (NAC) (a ROS inhibitor; #HY-B0215, MCE), followed by 24 h of treatment with H_2O (control) or 100 μM TBHP. (B) Immunofluorescence assay of NPCs showing representative confocal images of untreated cells or cells treated with 100 μM TBHP for 24 h and subjected to DAPI (blue), the mitochondrial marker COX IV (red), and KEAP1 or PGAM5 (green) stains, respectively. Overlays were displayed in yellow. Scale bar = 20 μm/2 μm. (C, D) Concentration course analysis of the relative mRNA expression of KEAP1 (C) and PGAM5 (D) in NPCs treated with TBHP for 24 h. (E) Transmission electron microscopy (TEM) images of NPCs treated with H_2O (control) or 100 μM TBHP for 24 h. Scale bar = 2 μm. (F-I) Quantitative PCR analysis (F, G) and Western-blot assay (H, I) of KEAP1 and PGAM5 expression in NPCs. (J) Cell viability of NPCs pretreated with KEAP1 siRNA, PGAM5 siRNA, or NC siRNA transfection before TBHP exposure, as detected by CCK-8 assay. The mean ± SD is shown by the error bars. NC: negative control. NS: not significant.

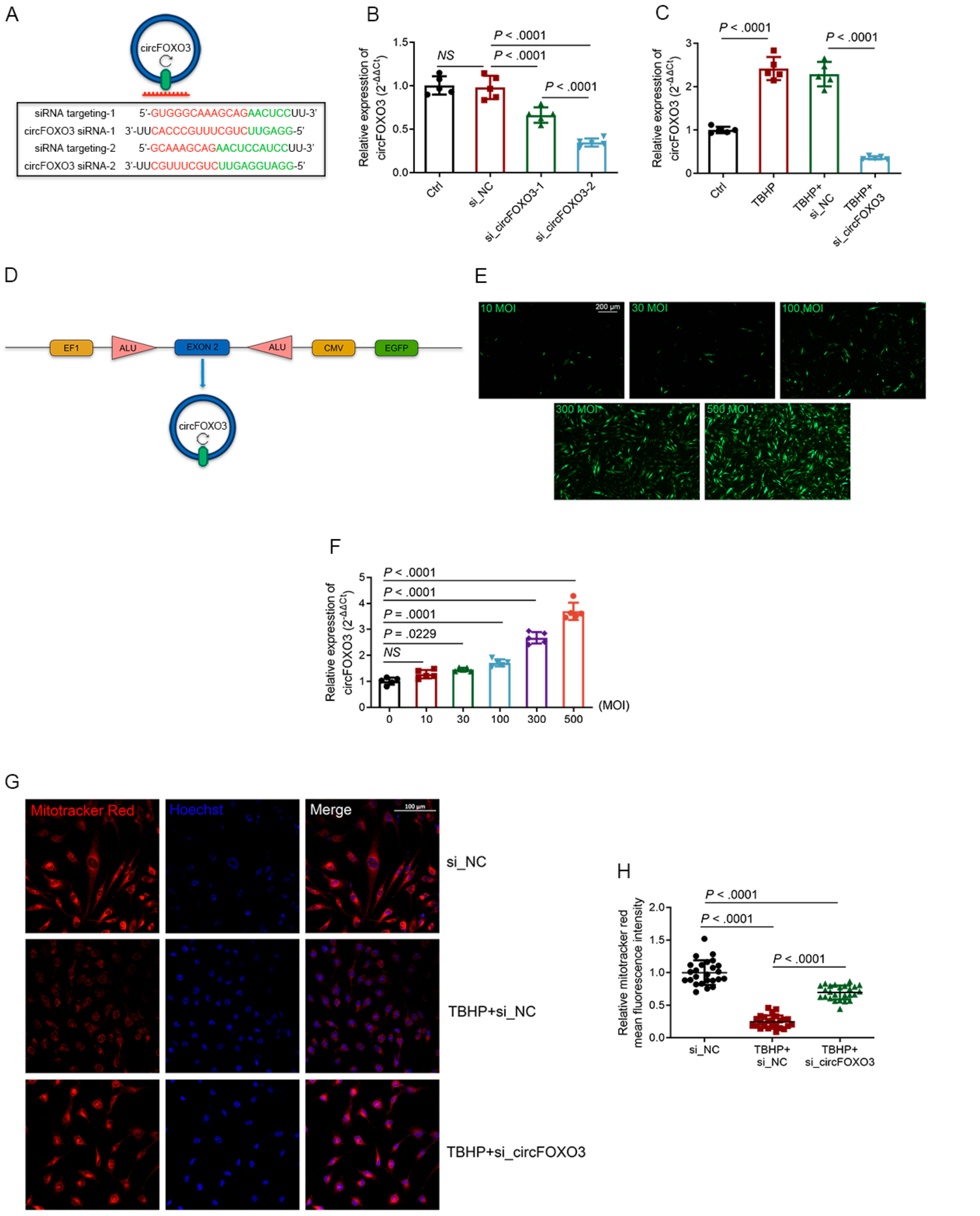

Supplementary Figure S3. The interference efficiency of circFOXO3 expression in NPCs.

(A) Schematic representation of the small interfering circFOXO3 vector structure. (B, C) Quantitative PCR analysis showing the expression levels of circFOXO3 in NPCs. (D) Schematic representation of the circFOXO3 overexpression vector structure. ALU: Complementary ALU pairs are required on opposite sides

of the back-splice to form circular RNA; EF1/CMV: A common promoter in vectors. (E, F) NPCs were infected with adenovirus at multiplicity of infection (MOI) 10, 30, 100, 300, and 500. Scale bar = 200 μm (E). Infection at MOI 500 showed superior efficiency compared to MOI 100 and 300 (F). (G, H) NPCs stained with Mitotracker Red (red) for mitochondria and Hoechst (blue) for nuclei. Scale bar = 100 μm (G). Mean Mitotracker Red fluorescence intensity was calculated using ImageJ software (data from 75 cells from 3 individual experiments), and the fluorescence intensity of the NC siRNA-transfected group cells was set as 1 for normalization (H). The mean ± SD is shown by the error bars. NC: negative control. NS: not significant.

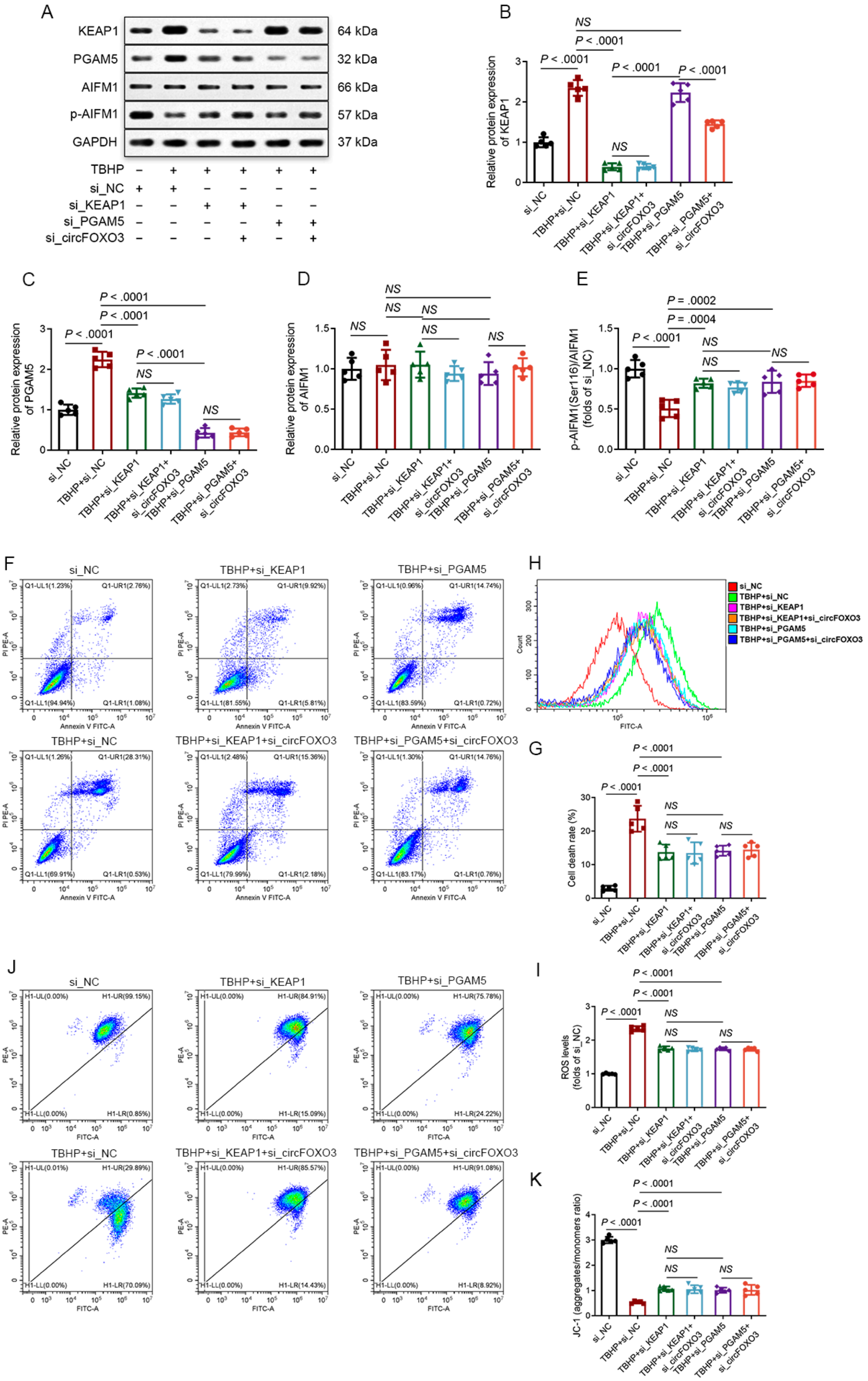

A
KEAP1
PGAM5
AIFM1
p-AIFM1
GAPDH
64 kDa
32 kDa
66 kDa
57 kDa
37 kDa
TBHP
si_NC
si_KEAP1
si_PGAM5
si_circFOXO3
B
Relative protein expression of KEAP1
C
Relative protein expression of PGAM5
D
Relative protein expression of AIFM1
E
p-AIFM1(Ser116)/AIFM1 (folds of si_NC)
P < .0001
P = .0002
P = .0004
NS
si_NC
TBHP+si_NC
TBHP+si_KEAP1
TBHP+si_KEAP1+ si_circFOXO3
TBHP+si_PGAM5
TBHP+si_PGAM5+ si_circFOXO3
F
PI PE-A
Annexin V FITC-A
H
Count
FITC-A
TBHP+si_KEAP1+si_circFOXO3
TBHP+si_PGAM5+si_circFOXO3
G
Cell death rate (%)
J
PE-A
FITC-A
I
ROS levels (folds of si_NC)
K
JC-1 (aggregates/monomers ratio)

Supplementary Figure S4. Knockdown of KEAP1 and PGAM5 counteracts the protective effect of circFOXO3 knockdown on oxeiptosis in NPCs.

(A-E) Western-blot analysis showing the protein expression levels of KEAP1, PGAM5, and AIFM1 phosphorylated at Ser116 in NPCs. (F-K) Flow cytometry analysis of Annexin V/PI (F, G), DCFH-DA (H, I), and JC-1 (J, K). The mean ± SD is shown by the error bars. NC: negative control. NS: not significant.

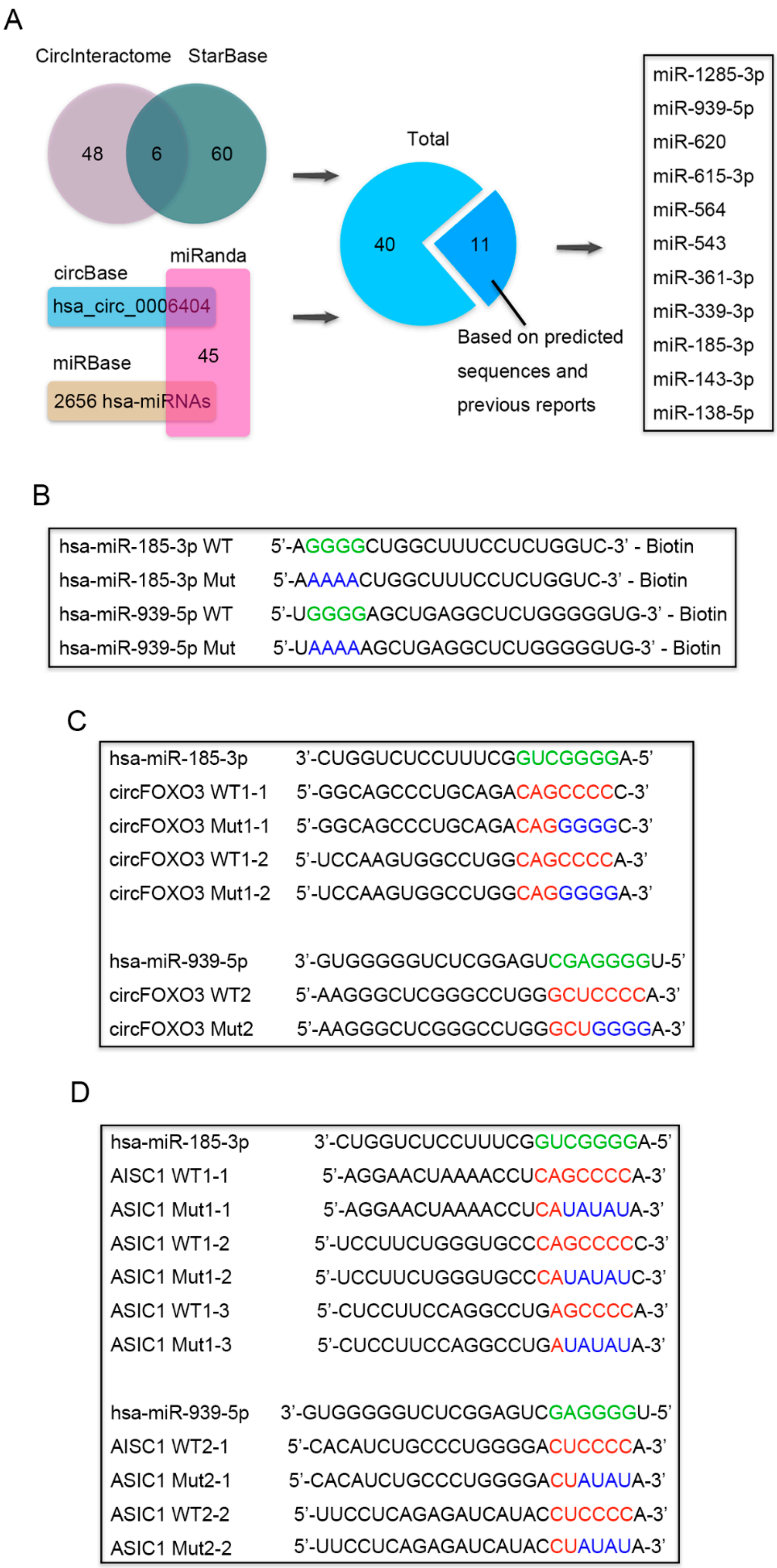

Supplementary Figure S5. MiRNA screening and targeted sequence information.

(A) CircFOXO3-targeting miRNAs were predicted based on the intersection of the CircInteractome and

StarBase databases. Additionally, miRanda matched and predicted 2656 miRNA sequences from the miRBase database with the hsa_circ_0006404 (circFOXO3) sequence from the circBase database. (B) Biotinylated wild-type (WT) and mutant (Mut) miR-185-3p or miR-939-5p sequences which were utilized in the RNA pulldown assay. (C, D) Predicted matching sequences of circFOXO3 (C) or 3'UTR of ASIC1 with miR-185-3p/miR-939-5p. WT and Mut luciferase reporter plasmids were utilized in dual-luciferase reporter gene assay.

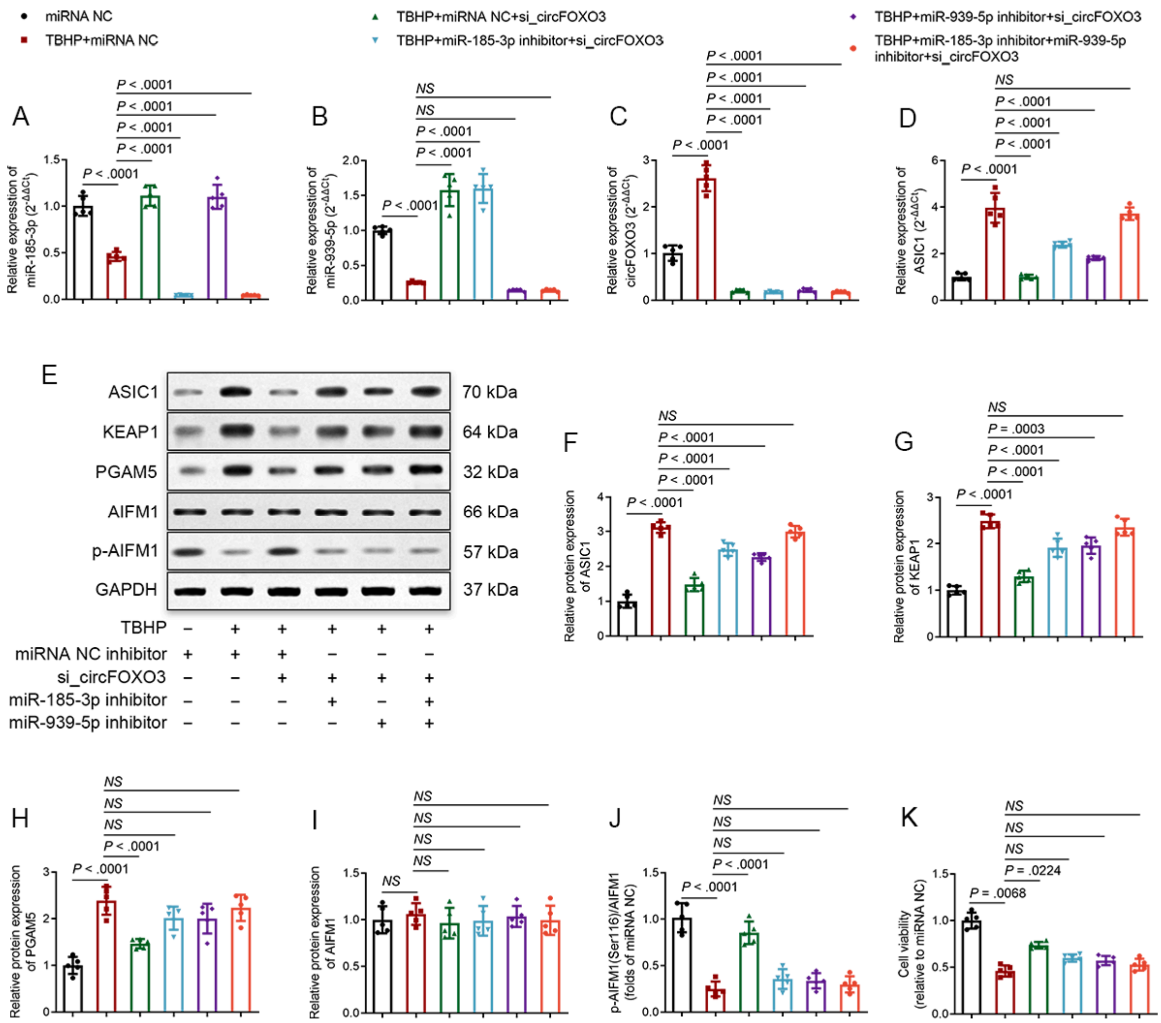

Supplementary Figure S6. The rescue experiment confirmed the existence of the circFOXO3-miR-185-3p/miR-939-5p-ASIC1 axis in NPCs

(A-D) Quantitative PCR revealed RNA levels of the circFOXO3-miR-185-3p/miR-939-5p-ASIC1 axis in NPCs treated with TBHP and circFOXO3 knockdown alone or along with miRNAs knockdown. (E-J) Western-blot assay showing the protein expression levels of ASIC1, KEAP1, PGAM5, and AIFM1 phosphorylated at Ser116 in NPCs. (K) The cell viability of NPCs treated with TBHP and circFOXO3 knockdown alone, or along with miRNAs knockdown, was detected by CCK-8 assay. The mean ± SD is shown by the error bars. NC: negative control. NS: not significant.

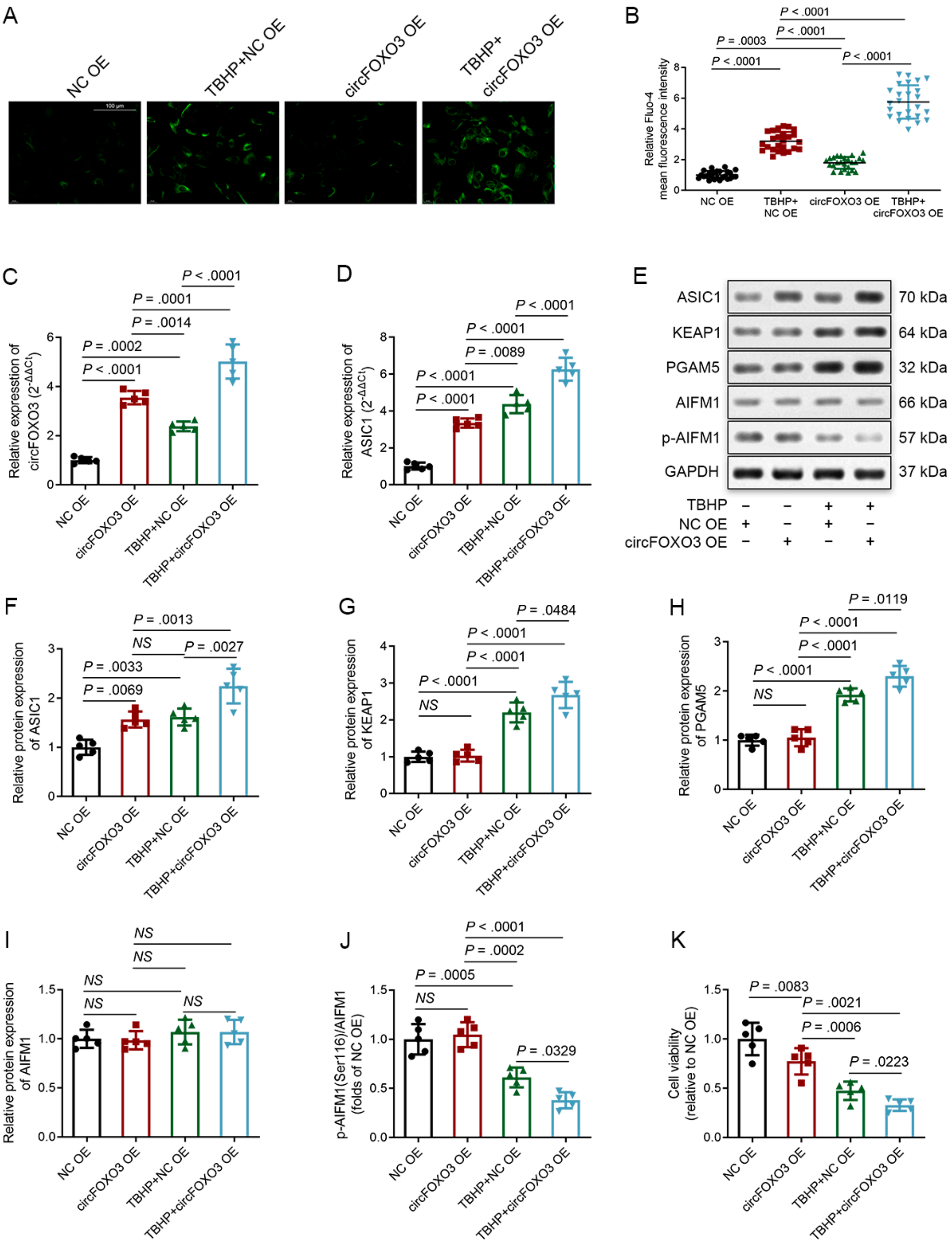

Supplementary Figure S7. Overexpression of circFOXO3 increases the susceptibility of NPCs to oxeiptosis

(A, B) Ca^{2+} levels in NPCs were detected using Fluo-4 (green). Scale bar = 100 μm (A). The mean fluorescence intensity of Fluo-4 was calculated using ImageJ software (data from 100 cells from 3 individual experiments, respectively), and the fluorescence intensity of the NC OE group cells was set as 1 for

normalization (B). (C, D) Quantitative PCR analysis of circFOXO3 (C) and ASIC1 RNA (D) expression levels in circFOXO3-overexpressing NPCs treated with or without TBHP. (E-J) Western-blot analysis of ASIC1, KEAP1, PGAM5, and AIFM1 phosphorylated at Ser116 protein expression in NPCs. (K) The cell viability of NPCs was detected using CCK-8 assay. The mean ± SD is shown by the error bars. NC: negative control. OE: overexpression. NS: not significant.

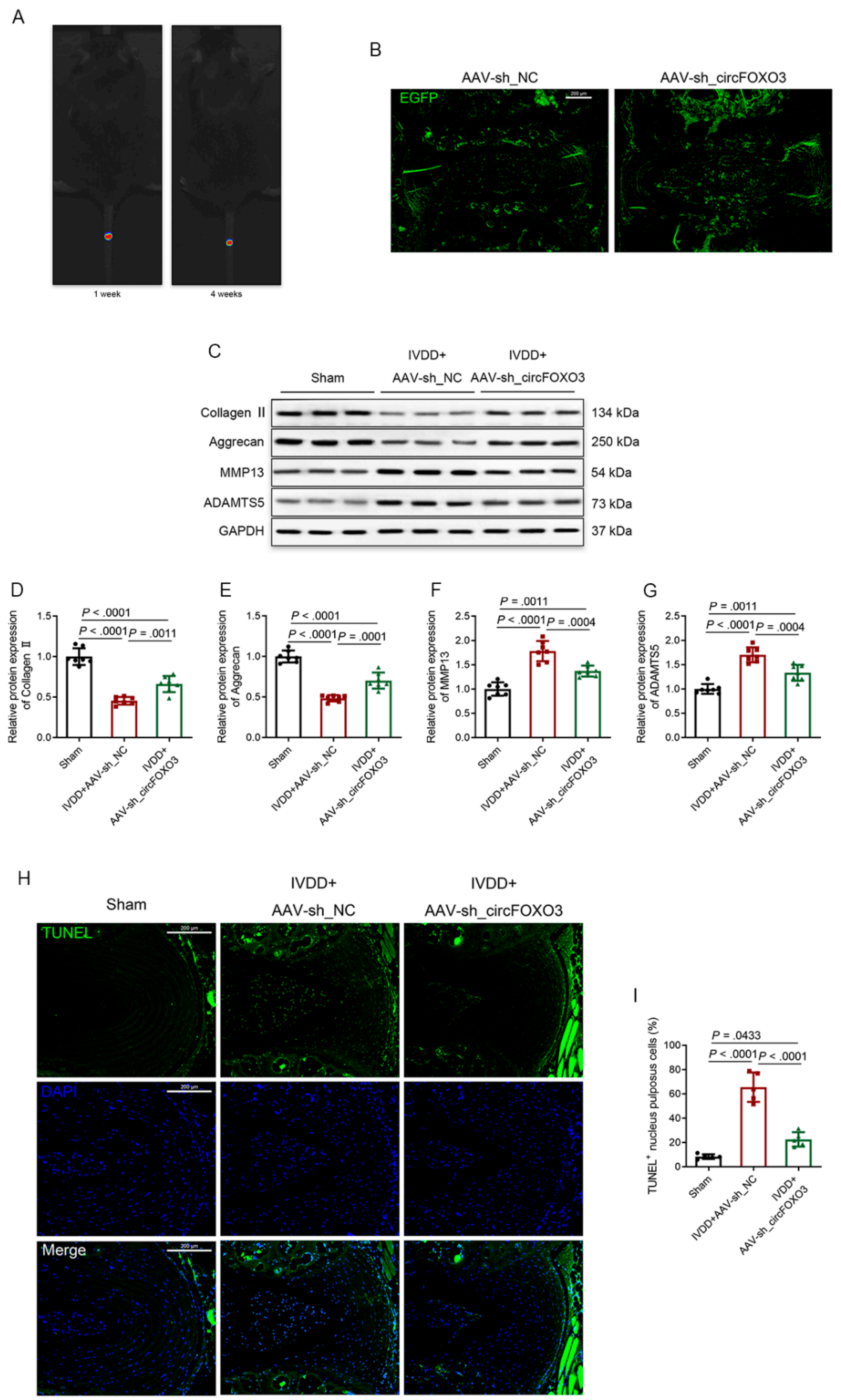

Supplementary Figure S8. Additional in vivo experimental results.

(A) In vivo bioluminescence image in mice at 1 week and 4 weeks after the administration of fLuc-labeled AAV-sh_circFOXO3. (B) Fluorescence imaging post intradiscal injection of EGFP-labeled AAV-sh_NC and

AAV-sh_circFOXO3. Scale bar = 200 µm. (C-G) Western-blot analysis of Collagen II, Aggrecan, MMP13, and ADAMTS5 protein expression in NP tissues of each group. (H, I) Representative terminal deoxynucleotidyl transferase dUTP nick end labeling (TUNEL) staining images of mouse annulus fibrosus (AF) punctured intervertebral discs. Scale bar = 200 µm (H). The statistical analysis of TUNEL-positive NPCs in the intervertebral discs of each group (I). The mean ± SD is shown by the error bars. NC: negative control. NS: not significant.

Table S1. Primers list for qPCR.

Target	Forward primer, 5′-3′	Reverse primer, 5′-3′
Human		
hsa_circFOXO3	GGGGAACTTCACTGGTGCTAAG	GACCCGCATGAATCGACTATG
hsa_circFOXO3 (convergent primer)	TCAGGCTCCTCCTTGTACTCAACT	TGTCACATTCCAAGCTCCCATT
GAPDH	GTCTCCTCTGACTTCAACAGCG	ACCACCCTGTTGCTGTAGCCAA
KEAP1	CGACGGTTCTACGTCCAGGC	GACTGTCGGAAGTAGCCGCC
PGAM5	CCTGGCAAGCTTGGGGTTGA	ACTGTCCTCCTCCTGCCTGG
ASIC1	CAGACGTGGAAAGTGCCAGA	GCTCTCGCAGGGATTGTGT
COMT	CGCTTCTCTTGGAGGAATGTG	CAGGAACGATTGGTAGTGTGTG
IGF1R	ACAATGAGTGCTGCCACCCC	AAGTCACGGTCCACACAGCG
MECP2	CCTGGGCGGAAAAGCAAGGA	CGGGCTGAGTCTTAGCTGGC
MAPK1	TCGAGCACCAACCATCGAGC	GGTGGTGTTGAGCAGCAGGT
HMGA1	CAGCGAAGTGCCAACACCTAA	GTTGTGGTGGTTTTCCGGGT
SOX4	ACAGGGCGGCTGGTTAATATCTC	TGGACACTGGTGGCAGGTTAAG
ERBB2	CGAGTACTTGACACCCCAGG	CTTTGAAGGTGCTGGGTGGA
TSC2	GCAACGACTTTGTGTCCATTGTCTA	GGACACCAGGTTGCACTCGTA
U6	CGCAAGGATGACACGCAAATTC	
hsa-miR-138-5p	AGCTGGTGTTGTGAATCAGGC	
hsa-miR-143-3p	CCTGAGATGAAGCACTGTAGC	

hsa-miR-185-3p	GCTGGCTTTCCTCTGGTCAA	
hsa-miR-339-3p	CTCGACGACAGAGCCGAAA	
hsa-miR-361-3p	TCCCCCAGGTGTGATTCTGATTT	
hsa-miR-543	AAACATTCGCGGTGCACTTCTT	
hsa-miR-564	ACGGTGTCAGCAGGCAAA	
hsa-miR-615-3p	AGCCTGGGTCTCCCTCTTAA	
hsa-miR-620	GCGCGCATGGAGATAGATATAGAA	
hsa-miR-939-5p	TGAGGCTCTGGGGGTGAAA	
hsa-miR-1285-3p	TCTGGGCAACAAAGTGAGACCT	
Mouse		
Mmu_circFOXO3	CTGGTGCTAAGCAGGCCTCA	GCCTTCATTCTGAACGCGCA
GAPDH	TTCCAGTATGACTCTACCCACGGCA	GCACCAGCATCACCCCATTTG
ASIC1	GGTCTTCCCAGCTGTCACCTTC	ACTCTCATCCAACCCGAGCA

Table S2. Sequences of siRNAs.

SiRNA	Sense, 5′-3′	Antisense, 5′-3′
si_KEAP1	GUGCUGUCAUGUACCAGAUUU	UUCACGACAGUACAUGGUCUA
si_PGAM5	GAGGACAGUUACGAGAUCUUU	UUCUCCUGUCAAUGCUCUAGA
si_hsa_circFOXO3-1	GUGGGCAAAGCAGAACUCCTT	GGAGUUCUGCUUUGCCCACTT
si_hsa_circFOXO3-2	GCAAAGCAGAACUCCAUCCTT	GGAUGGAGUUCUGCUUUGCTT
si_NC	UUCUCCGAACGUGUCACGUTT	ACGUGACACGUUCGGAGAATT

Table S3. Sequences of mimic and inhibitor of miRNAs.

MicroRNA	Mimic, 5′-3′	Inhibitor, 5′-3′
hsa-miR-185-3p	AGGGGCUGGCUUUCCUCUGGUC	GACCAGAGGAAAGCCAGCCCCU
hsa-miR-939-5p	UGGGGAGCUGAGGCUCUGGGGGUG	CACCCCCAGAGCCUCAGCUCCCCA
miRNA NC	UUCCCCGAACAACAACGUGUCACCACC	-

Table S4. Sequences of RNA FISH probe and AAV-sh_circFOXO3.

Items	Sequence, 5′-3′
RNA FISH probe	UUGUGCCGGAUGGAGUUCUGCUUUGCCCACUUC
AAV-sh_circFOXO3	CCGGGAAATGGGCAAAGCAGAACTCTCGAGAGTTCTGCT TTGCCCATTTCCCTTTTTG
